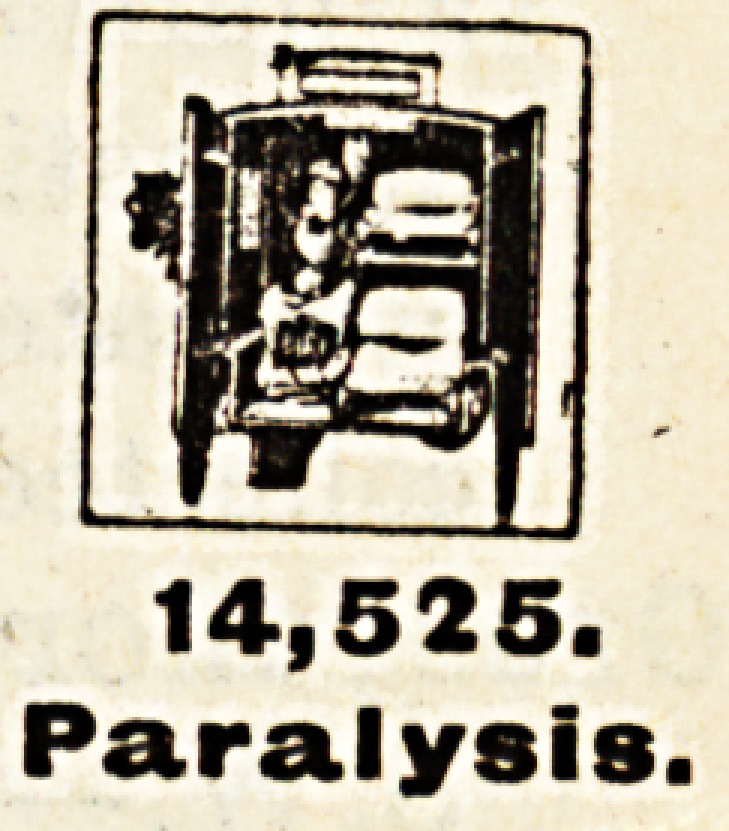# Special Hospital Sunday Supplement

**Published:** 1916-06-24

**Authors:** 


### The Special Hospital Sunday Supplement.

The Hospital, June 24, 1916.

### HOSPITAL SUNDAY, 1916.

Hospital Sunday in 1916 should be regarded by the clergy and ministers of all denominations throughout the Metropolis, and indeed throughout the Empire, as the greatest day each one of them has probably lived to see in the history of their ministry. The Great War has come, and we as a nation are taking part in it because we believe in our hearts it is God's work to defend the weak, the smaller nationalities, the innocent women and children, with the feebler nations, and to resist with all our might the great, organised, long-worked-for, and deliberate attempt of one ambitious nation to establish over all others who do not submit to its will the tyranny of brute force in its most relentless, murderous, and cruel form. That nation by its actions so far has seemed to court, if it has not even secured, the co-operation and continuous help of the forces of evil which seem to egg on the great aggressor nation to further infamies, not the least of which has been the deliberate and murderous slaughter of tens of thousands of men of their own race and nation during the awful and persistent conflicts at Verdun. Is there a single minister of God in the whole country that can think without a shudder of the awful responsibility and dreadful reckoning which William the Second of Germany will one day have to face? It follows from all this that every bishop, priest, clergyman, and minister throughout the land, representatives of whom have shown commendable courage in ministering at the Front to the wounded and the dying, must recognise the high privilege every one of them will have when they stand in their pulpits on Hospital Sunday. Before that day they must face the responsibility of deciding to advocate to the utmost of their power the claims of our hospitals, or to let the opportunity pass. Every preacher will stand before God in the face of his congregation on Hospital Sunday, 1916, as the mouthpiece of the nation, to give expression to the unanimous admiration and gratitude of everybody for our brave sailors and soldiers who are fighting the battle of right and sacrificing their lives in God's service in this terrible war.

What has the wounded warrior to rely upon when he is smitten and grievously wounded in the fight? Is it not the service of the ambulance and medical staff who first of all tend and remove him by stages ultimately to the base hospital from which he is brought with surprising speed and efficiency to one of the great hospitals in the homeland? The calls upon the metropolitan hospitals, as upon all voluntary hospitals throughout the country, in consequence of the war, have been well-nigh overpowering. The claims and needs of the voluntary hospitals are surely paramount. Those needs are greater under war conditions than normally, for not only have upwards of one-third of the available beds been placed at the disposal of the Government for wounded warriors, but the drain for workers at the war upon the medical, surgical, and resident medical staffs, and the matrons, sisters, and nurses, has been continuous and increasing. War prices and war difficulties with higher rates of remuneration to employees have added materially to hospital expenditure. These are incontrovertible facts and every preacher has the privilege of giving them the widest and most convincing publicity from his pulpit on Hospital Sunday. We hope every minister of religion throughout the land will give of his best in his endeavour to help the hospitals to help the people and the Government in existing circumstances.

So much for the necessary and material facts. The means and will to do good deeds make good deeds done. May God move the hearts of the clergy of all denominations to make the effort of their lives to secure the provision of all these means for the voluntary hospitals in 1916. But there is one other matter in connection with the war which touches the leaders of religious thought throughout the land more nearly and closely in this connection than anything that has ever happened so far in the history of the world. This is the spiritual awakening of the men of all classes throughout our nation and Empire, the bravest, the strongest, and the best, who have come forward to join the Forces in numbers so great as to make other nations marvel, and every man amongst us praise God for the awakening which has come to our race in these days of supreme trial which the Great War has created. The spirit of our sailors and soldiers, the courage they exhibit, the cheerfulness and extraordinary patience with which they have faced the most cruel trials, the grievous disasters that can happen to the physical well-being of man, and the shouldering of the burden of resistance to evil which this war has called forth, should surely find a closer, and a deeper, and more direct echo in the hearts of the clergy and ministers of all denominations than it does even in that of the ordinary man and woman in our midst?

Is this sentiment to exhibit vitality and action on the part of the clergy? The pulpits on Hospital Sunday will testify, and the results should, with God's blessing, prove as remarkable as has been the uplifting of the nation in defence of the right at the call of the weak, the defenceless, and the wronged. The spiritual awakening of our race at this time of war has produced a revolution in feeling, and as we believe it will be exhibited in action, in the overwhelming majority of men and women throughout the nation and Empire. We could wish that among all Churches and creeds this awakening to spiritual things should manifest itself now in the formation of Guilds of the House of God, that the recognition of God's House and His abiding Presence therein may fill the heart of the nation with reverence, and destroy for ever the sleepy, lifeless, monotonous, commonplace, perfunctory performance of services therein which, alas! has in many places seemed so to affect the clergy and ministers as to shake their faith and cause them to lose touch not only with individual members of their congregations, but with the spirit of worship.

To-day is the opportunity for the clergy by co-operating to bring life and energy and reverence, earnestness and purpose into religious service in every House of God once more. This atmosphere of the predominant force present must strike every worshipper who enters the House of God in our nation and Empire. The presence of the Spirit as it is manifested throughout our affairs, and especially in the trenches and in face of the enemy, is a wonderful manifestation of supreme importance. The opportunity of the home clergy and ministers at present is to spiritualise themselves, God's House, and His worship for the return of our warriors from the Front. Will it not be terrible indeed if the soldiers at their home-coming miss the presence of the Spirit impressed upon minister, congregation, and service?

In this connection a sermon by the Rev. John Penfold (see page 289), which was preached since this article was written, cannot fail to yield helpfulness to all earnest and devout people.

### THE BED-ROCK OF THE SUNDAY FUND MOVEMENT.

In this, the second summer since the war, Hospital Sunday is faced with the task of maintaining its position now that the first great wave of charity has spent itself. If the old record be maintained, as it must be, it will be because the public recognise the new need, and not because fresh arguments or sensational appeals are attempted to move them. We in England, who have not yet known the reality of invasion, have come more slowly than our Allies to personal grips with the oppressive reality of war. It is only now that the sense of strain is beginning to come home to many of us. The first draught on our enthusiasm has been met. The first draught on our endurance is now beginning. This personal experience, for there can be none now who has not felt it, is reflected in practical terms in our hospitals also, and that is the real reason why this year more than ever an effort must be made to maintain the great Funds, and especially the Metropolitan Hospital Sunday Fund, at full strength.

Shortly after the declaration of war, the voluntary hospitals opened their beds to the wounded soldiers so freely that a warning had to be uttered on behalf of the civil population remaining at home. That warning has been justified by time, for while the claims of the wounded have continued to be insistent, it has been found quite impossible further to curtail the hospitals' services to the men, women, and children who ordinarily resort to them. We are told that our Army has been recruited by 5,041,000 volunteers. We may roughly estimate the population at 40,000,000. These figures, set side by side, show in one instant how impossible it is for the ordinary demands on the hospitals to be diminished. Once the clergy grasp a fact of this kind, the direction and force of their appeals on behalf of Hospital Sunday can be guaranteed; for the necessity is unescapable.

### The Clergy's Own.

It must never be forgotten that among the many appeals which issue from the Churches in the course of the year, that on behalf of Hospital Sunday has the peculiar quality of being the clergy's own. For the idea which has expanded into the Hospital Sunday movement emanated from one of themselves, the late Canon Miller, who was the real founder of the enterprise. It has proved so successful that the Sunday Fund is now faced with numerous rivals, who have traded on its original principle of devoting one day in the year to the collection of funds on behalf of a special object. The various Rose Days, Ribbon Days, and Flag Days are nothing but derivations of Hospital Sunday, and no one should subscribe to them without remembering whence they have drawn their ideas, and which day is the parent of these others.

But there is still another reason why the clergy, in whose hands the success of the Sunday collections largely rests, should make the most of the privilege accorded to them as preachers on behalf of the Sunday Fund movement. Many of them have had experience of the fighting; some have even shown such a martial spirit as to prefer the soldier's uniform to the cassock and surplice of the Prince of Peace. They have witnessed, perhaps aided in, the work of destruction. It is only by helping the hospitals and the cause of charity that they can maintain also the work of reconstruction, and keep the flag of civilisation flying behind, if not actually on the battlefield.

### How War Lords are Punished.

In the larger sense this is the work of the hospitals, to undertake works of mercy on the one hand, and to subdue science to the service of humanity on the other. Until a man has learnt to feel the same pride in the latest achievement of science and medicine as he does in the latest big gun he is still a grown-up child and half a savage. Luckily the importance of this attitude is better recognised now than it was in July 1914. For, while it is possible for insane people to desire war in time of peace, war itself soon cures them of their delusion. There is no better tonic than a knowledge of facts, and no better education than a grasp of what they stand for. It is these which lead men to support great movements; for action is only a function of thought, and generosity only the immediate translation of intelligence and feeling into action.

### THE NEW REDEMPTION OF THE WORLD.

### Our Warriors, the Churches, and Ourselves.

Since the first article in this Supplement was written a sermon has been preached by the Rev. John Penfold, incumbent of St. James's Church, Guernsey, which follows. We could wish that it might be preached in every place of worship throughout the country and the Empire. It indirectly raises the question, Will the Churches be alive and prepared for the return of our warriors full of the Spirit and reconciled to Almighty God? If they are not, the results may prove as disastrous to the Churches as they will be lamentable and onerous to all Christian men and women. May all the Churches become alive and filled with the Spirit.

### The Meaning of National Service.

Philemon 15.—"Perhaps, therefore, he was parted from thee for a time that thou mightest have him for ever." The mystery of the Ascension is the withdrawal of our Lord into the invisible world, that He might reinforce the lives of His disciples with all the resources of that world. Ascensiontide sets our minds at work in the direction of those vast tracts of being which are beyond our mortal ken. It reminds us that they are just as real as these near-by things which we touch and see and handle every day. When Jesus Christ vanished from the sight of His Apostles, they knew, and we know, that He did not disappear into empty space and "like the aery fabric of a vision, leave not a wrack behind." He became in a mysterious but effective way nearer to His own than ever. His Ascension was realised by them as the beginning of a new relationship, in which His influence was even more close and intimate, and fellowship with Him even more active and vital than in the days of His flesh. The coming of the Holy Ghost was not a substitute for the Presence of Jesus Christ. It was a more complete consciousness of that Presence in power.

That was why in the Early Church people were able to do wonderful things. That they really did them is matter of history, so that men who themselves professed to be wonder-workers, like Simon Magus and Elymas, were impressed and were ready to give large money to Peter the Galilean fisherman and Saul the Jew of Tarsus. They did them because they lived in constant touch with the spiritual, even as their Master had said, "The works that I do shall ye do also, and greater works than these shall ye do, *because I go to the Father*." The fact that He reigned for them in the spiritual world did not take Him away from them. It brought that world nearer to them, and made it theirs, so that all its resources were behind them. "I can do all things, through Christ which strengtheneth me." Conscious of the spiritual forces at their back, they could, and did, go anywhere and do anything, so that a few peasants, mourning a lost Leader, were transformed into men who wrought so mightily that they were afterwards described by their critics as "these men which have turned the world upside down." They were not terrified by threats, they were not put off by difficulties, they did not fear failure, they were not afraid of death. They might have said with more conviction than men of a later day, as they looked up to God, "Where Thou art Guide, no ill can come."

It is one of the blessings of this war that it has brought many of us nearer the invisible world and the powers of Heaven. We are seeing strange and terrible things. Things we trusted in have failed us. Our money, of which we made so much, our elaborate civilisation, our votes for which our political leaders were ready to promise anything, even a new heaven and a new earth, our political system which we had exalted into a kind of faith. All these things have failed to save us, while grim facts we never thought to look upon stare us in the face. One thing in particular has forced itself upon us which has made it impossible for things ever to be the same again.

Before the war, and after it, right up almost to the present hour, we have clung persistently to an error which we tried to make respectable by giving it a fair-sounding name. That is characteristically British to cloak an ugly thing with a fine phrase. This error we called "The Voluntary Principle." It sounds so well, does it not? But what we meant by it was that even in the greatest things a man's life consists in doing what he likes, and that even in the greatest crisis we must safeguard the right of every citizen to do as he likes. Suddenly we have changed all that, and, except a noisy few, we have accepted the change in silent acquiescence. The State has claimed that the citizen must give himself up to its service whether he likes or not, and we have all willingly endorsed that claim. And we are *sending*, not asking them if they will go, but *sending* thousands upon thousands of our fellow-citizens to undergo hard discipline, to undertake hard duty, to venture their bodies at the risk of wounds and death, in order to redeem their country, to suffer and to die in order that others who come after them may live lives of freedom and happiness after they are dead. That is the meaning of National Service. The voluntary principle meant that the unselfish, generous, and brave may die in order that the selfish and the cowardly may live. But National Service means that the citizen must be prepared, if need be, to die in order that the Empire may live and be free. "He that will keep his life shall lose it; he that will lose his life for My sake and the Gospel's [that is, for the highest end] shall keep it unto life eternal."

That is what the war means for us all now, the life well lost—a new redemption of the world through sacrifice. Our race is being redeemed through the blood of our brothers and sons. Well may we write their names on rolls of honour and commend their souls to God, for they have rekindled in Britain a flame that, God forgive us, had begun to wax dim, but which, by Christ's grace, shall never fade again; the shining light of redemption through sacrifice of the life given to save. That light shines out of the spiritual world "where Christ is seated at the right hand of God," where, He has told us, "there shall be no more death." Only in the reality and supremacy of that world does the claim of that life find foundation and justification. How dare we claim a man's life from him if this world be all? Standing on higher ground, seeing with clearer vision, we bid him to live and die that he may live for ever the life that is hid with Christ in the eternal.

How this truth comes home as we think of the great sea battle on the eve of Ascension Day! Little we imagined, we who were here in church early on Ascension morning, asking God, as we knelt before His altar, to remember for good those who were serving us by sea and land, little dreamed we that while the words were on our lips the air was still quivering with the roar of the great guns, the sullen waters were still swirling over the places where the fine ships had gone down, bearing with them the unshrouded bodies of brave men to lie in the unrecorded deep until the sea shall give up its dead. All through Ascension Eve our seamen were fighting against odds to redeem their country. And when the day broke and the ships sheered off, was it all at an end? Did they as fools die, giving up for nothing all the promise of the years? Or is not theirs the good part that shall not be taken away, the inheritance of the spiritual world, the prize of those who will lose their life to keep it unto life eternal, relying on His Word Who left us this message for Ascension Day, "I ascend unto My Father and your Father, and your God and My God"? The spiritual world alone gives meaning and cohesion to the things of this fleeting world of sense. Truly there is no force or value or meaning in the messages of the Gospel, no reality of love, no compelling call to duty, apart from that world which our Lord opened to us at His Ascension into Heaven.

"There shall no evil happen unto thee." Can we attach any meaning to that as we think of perhaps five thousand of the best men of Britain lying to-day in their vast and wandering grave? The secret lies in the spiritual world which is about us, from which our Lord helps us. "Hold thou me up, and I shall be safe." We turn the eye of Faith towards the prospect of Eternity, "and out of darkness come the hands that reach through Nature, moulding men." Death, jealous death, attempts to bar the road, defeat and apparent failure strive to blur the vision; but, "the righteous shall hold on his way, and he that hath clean hands shall be stronger and stronger."

### SHAKESPEARE'S MEDICAL PLAY.

Even the greatest war in history has not quite overshadowed the interest in the three hundredth anniversary of the death of Shakespeare, for, like the war, the great poet enters into the lives of us all. Every student of his plays will recall passages or characters that reflect Shakespeare's interest in the medical lore and customs of his day. His medical characters are few and varied. To Cerimon in "Pericles" is paid this tribute which might be repeated in modern life:

"Your honour hath through Ephesus pour'd forth
Your charity, and hundreds call themselves
Your creatures, who by you have been restor'd."

In "Merry Wives" the physician is a Frenchman! His ill-tempers are part of the comedy. In "Cymbeline" the doctor protects Imogen by giving her an opiate instead of a poison. The physician in "Lear," to whom Cordelia appeals to cure her father's madness, gives a reply which could hardly be bettered to-day:

"There is means, madam:
Our foster-nurse of nature is repose,
The which he lacks; that to provoke in him
Are many simples operative, whose power
Will close the eye of anguish."

There is no more dignified figure in the plays than that of the doctor who watches Lady Macbeth in her sleep-walking. In her guilt he sees the universal need, "God forgive us all," and recognises that there are some problems beyond the power of the healing art:

"More needs she the divine than the physician."

Much more frequent than the introduction of actual physicians in the plays are the references to the homely medicines of the time. "Flax and white of egg for a bleeding face," "Cobwebs for cuts," "Spermaceti for burns," and so on.

But one play, "All's Well that Ends Well," may actually be called a medical play, inasmuch as the plot turns upon the efficacy of a physician's prescription. A well-known physician has died and left to his daughter a number of prescriptions, amongst them one, "the darling of his old experience," which was a cure for the very disease (malignant it is called) from which the King is dying, and for which his own physicians could find no cure. Helena obtains an audience of the King and persuades him to try the remedy.

The interest of the play from the medical point of view is practically in one scene in which Helena has her interview with the King. She is introduced as "Doctor She," and the old lord who introduces her pays her a compliment which might justly be paid to many of her modern successors:

"One that in her sex, her years, profession,
Wisdom and constancy hath amazed me more
Than I dare blame my weakness."

She tells of her father's bequest to her and begs the King to let her apply the remedy to his disease. At first he refuses. Something very like medical etiquette is urged as well as the strangeness of the proposal. The real greatness of the scene is in the recognition on both sides that it is more than a mere question of obeying a written prescription. Helena asks that the King will not let her ignorance or unimportance blind him to the possibility of her method being the means which God deigns to use for his restoration.

"He that of greatest works is finisher
Oft does them by the weakest minister.
* * * *
Oft expectation fails and most oft there
Where most it promises, and oft it hits
Where hope is coldest and despair most fits.
* * * *
Dear sir, to my endeavours give consent;
Of Heaven, not me, make an experiment.
I am not an impostor that proclaim
Myself against the level of mine aim;
But know I think and think I know most sure
My art is not past power nor you past cure."

In answer to the King's challenge Helena declares herself willing to submit to shame, torture, even death, if the remedy should fail. The King at last consents and is cured. The sequel of the story, which Shakespeare got from Boccaccio, has in it some exceeding repulsive features, but which are quite outside the scope of this article. It is of interest to remember that the greatest of all poets, who has been so much in the minds of all of us of late, associated one of his boldest studies of human character in Helena with that profession of healing which, too, is in all our minds just now. We are sure that if Shakespeare were living to-day he would plead for the support of our Homes of Healing which are open to all, and especially just now to our soldiers. It is not too much to say that we may even think of the great poet pleading that in *his* year the work of the hospitals may have a recognition such as never before in the history of the Hospital Sunday Fund.

Rev. W. Hardy Harwood.

### THE FOUNDER OF ST. BARTHOLOMEW'S HOSPITAL.

### The Preservation of Rahere's Tomb.

There are few churches in London more beautiful than the Church of St. Bartholomew the Great, in Smithfield, with its lovely Norman cloisters, surmounted by the equally beautiful clerestory of the Early English style. For close on eight centuries it has withstood the ravages of time and fire, sheltering beneath its roof the remains of Rahere, the builder of the church and the founder of St. Bartholomew's Hospital. His tomb lies on the north side of the altar, but is now invisible owing to its covering of sandbags to preserve it from aerial bombs. On the tomb lies an effigy of Rahere, showing the first canon and prior with shaven crown, in the habit of an Augustinian canon. At his feet a crowned angel holds a shield bearing the arms of the Priory, gules two lions passant guardant with two crowns. On each side of the prior is a small kneeling figure of a canon reading from a book. Over it is a vaulted canopy of the fifteenth century, whilst there are some panels of the same date on the base.

The preservation of this historical and magnificent Norman tomb is a labour of love to those in whose care it is, and, indeed, London and its medical profession would be poorer if any accident occurred to the tomb of one who, in the early history of the country and of medical science, did so much to create and foster a love for the poor in their afflictions. He was the first great benefactor to a hospital, and St. Bartholomew's Hospital bears testimony to his zeal and piety in that early Norman age.

Tomb of the Founder of St. Bartholomew's Hospital.

St. Bartholomew's is the oldest of our London hospitals, being founded in 1123, whilst St. Thomas's owes its foundation to the now forgotten Bermondsey Abbey of St. Saviour in 1213, a century later, and the Hospital of St. Mary of Bethlehem was inaugurated in 1246. There were, according to Stow, several lazar-houses in London for the reception of persons suffering from leprosy, these being the Lock, in Southwark, at the south end of the now notorious Tabard Street, another between Mile End and Stratford-le-Bow, another at Kingsland between Shoreditch and Stoke Newington, and another at King's Cross; but Rahere was the first to institute a hospital for the sick poor of London, and its doors have ever since been widely open for their relief and the amelioration of their sufferings.

In the Cottonian MSS. in the British Museum there is an account of the life of Rahere, written soon after his death by one who must have been intimately acquainted with the great founder, probably a monk of the establishment. The name Rahere is of Frankish origin, and occurs as a witness in the charters of the district of the eastern boundary of Brittany. The author says that which he writes was "testified to us that saw, and heard him, and were present in his works and deeds, of whom some have taken their sleep in Christ, and some of them be yet alive and witnesseth what we shall after say." Rahere, he says, was "a man sprung and born of low kinage, and when he attained the flower of youth he began to haunt the households of noblemen and the palaces of princes, where, under the very elbow of them, he spread their cushions with japes and flatterings, delectably anointing their eyes, by his manner to draw to him their friendship. And still he was not content with this, but haunted the king's palace, and among the more powerful press of that tumultuous court informed himself with polity and cardinal suavity, by the which he might draw to him the hearts of many an one. There in spectacles, in meetings, in plays, and other courtly mockeries and trifles intending, he led forth the business of all the day. Thus he was wise to the king and great men, gentle and courteous. This manner of living he chose in his beginning, and in the exercise of his youth."

This account of his early life in all probability was the reason why Stow, in his "Survey of London," describes Rahere as "a pleasant - witted gentleman, and therefore in his time called the king's minstrel."

But the Norman chronicler records a change in the life and aspirations of Rahere, who now "became foremost in repentance as he had been foremost in sin." His patron was Richard de Beaumes, Bishop of London, who devoted for some years the whole of the revenues of the bishopric to the rebuilding of St. Paul's. He was instrumental in the establishment of the Augustinians at the Priory of Holy Trinity, Aldgate, and with his great wealth founded the Priory of St. Osyth on the manor of Chich (St. Osyth Chick in Essex), where he died in 1128. He was an active, energetic worker for the Church, a good administrator, and a powerful follower of Henry I., who was afterwards the patron of Rahere. It was undoubtedly due to the friendship this early Bishop of London had for Rahere that secured for the latter in 1115 the prebendary stall of Chamberlayne's Wood in St. Paul's Cathedral.

About 1120 Rahere went on a pilgrimage to Rome, when he visited the place now shown as the martyrdom of St. Peter and St. Paul and the Three Fountains. He contracted malarial fever. "There at the shrine of the Blessed Apostles Peter and Paul," states the chronicler, "he, weeping upon his deeds, prayed to our Lord for remission of them. These two men lights of heaven, two men of mercy, Peter and Paul, he obtained as mediators. And while he tarried there he began to be vexed with grievous sickness, and his dolours, little by little, taking their increase, he drew to the extreme of life, which, dreading within himself that he had not satisfied to God, therefore he supposed that God took vengeance on him for his sins amongst outlandish people, and deemed that the last hour of his death drew nigh. This remembering inwardly, he shed out as water his heart in the sight of God, and all brake out into tears, that he avowed that

The Grand Staircase, St. Bartholomew's Hospital.

(The paintings are by Hogarth, who presented them to the Hospital A.D. 1736.)

if God would grant him health that he might return to this country he would make a hospital in recreation of poor men and minister to them the necessaries of life. And not long after the benign and merciful Lord beheld this weeping man, gave him his health, and approved his vow."

Rahere returned to London, where he was welcomed by his friends, to whom he explained his project, while he sought the assistance of the great-hearted citizens of London. They pointed out to him that the site was within the king's market, and supported by the Bishop of London, the barons and great men of the city, he approached Henry I., who was persuaded to grant Rahere authority to execute his purpose, and bestowed on him the title of the desired possession. At this time the whole of Smithfield was an open space, described by Stow as "moorish ground, which was therefore a common laystall of all filth that was to be avoided out of the city." It included the site of the Charterhouse, which was long the property of St. Bartholomew's Hospital before the Carthusians settled there. And now the founder commenced to build his hospital.

Rahere applied himself "to "the study and purification of the place, and decreed to put his hands to that holy building. He drew to him all the fellowship of children and servants, and with their help stones and other things profitable to the building was lightly gathered together. He played with them, in so much that he pleased the Apostle, through whose grace and help he raised up a great fane." He sought assistance by visiting other churches, priories, and abbeys, exhorting "multitudes of clerks and of the laity to follow and fulfil those things that were of charity and alms deed." Of his preaching it is recorded that "in this wise he delivered his sermon, that now he stirred his audience to gladness so that all the people applauded him, and then again he urged them to sadness and sorrow for their sins so that all the people were compelled to sighing and weeping." And it is added that "his life accorded to his speech, and his deed approved well his sermon." "The church," so the chronicler adds, "he made of comely stonework, table wise. And a hospital house, a little longer off from the church by himself he began to edify. The church was founded (as we have taken from our elders) in the month of March 1123. President in the Church of England William, Archbishop of Canterbury, and Richard, Bishop of London, who of due law and right hallowed a part of the adjoining field as a cemetery."

The first patient admitted to the hospital is recorded in the *Liber Fundacionis* as Adwyne of Dunwych, probably from Dunwich in East Suffolk, a countryman long settled in London. The hospital society consisted of a master and eight brethren of the Canons Regulars of St. Austin, and four sisters, who attended to the material needs of the patients.

Few of the early monasteries or abbeys ever obtained from any of the reigning monarchs a wider or more ample charter than that granted by Henry I. to Rahere, the founder of St. Bartholomew's Hospital. All its privileges were preserved, enlarged, and protected, and Henry, by taking the foundation under his own special protection, created a precedent which was followed by his successors—the kings and queens of England, who, whatever may have been their faults, ever preserved the rights and privileges granted to Rahere to this—our first London hospital for the sick poor. For it must not be forgotten that from the very beginning it was a hospital for the sick, and not a mere almshouse, and this is more explicitly set forth in a grant of privileges to St. Bartholomew's made by Edward III.

Rahere, states the chronicler, was desirous of proceeding to Rome to express his gratitude to the Pope for the charter, but was unable to make the journey owing

The Great Hall, St. Bartholomew's Hospital.

The Great Hall is in the North Wing of the present Quadrangle, which was built 1729-1758.)

"to growing impediments." It was evident from the account of his life that at this time Rahere must have felt old age and other infirmities overcoming his natural powers. There is no evidence to show what his age really was, but in 1137 he retired from the office of Prior and Master, and was succeeded by Hagno. A charter of this year is still preserved in which "Raherus sancti Bartholomei qui est in Smythfelde, prior," grants to Hagno the old Church of St. Sepulchre. He died in 1144.

### HOSPITALS AND THEIR SPECIAL NEEDS.

**Belgrave Hospital for Children, Clapham Road, S.W.** The Belgrave Hospital for Children is the only hospital for children in South-West London, and is situated in the midst of a densely populated neighbourhood of working people. In 1915 745 children were admitted, of whom 239 were under one year of age, and 32,115 attendances were made in the out-patient department. In view of the serious infantile mortality in this country, these figures bear eloquent testimony to the effort which is being made at this hospital to save the lives of the infants.

**British Hospital for Mothers and Babies, Woolwich.**—The special needs of this hospital at the present moment are, undoubtedly, funds and accommodation. While the bank account is overdrawn £300, the accommodation is utterly inadequate to the demands upon it. The number of beds is fourteen only, and the refusal of many applicants for admission, mostly the wives of sailors, soldiers, and munition workers, is most distressing to the management. A new hospital, with better accommodation for nurses and candidates for training, is urgently required.

**Canning Town Women's Settlement Hospital, Plaistow, E.**—With the larger hospitals crowded, additional responsibilities are thrown upon the smaller ones. The sick poor to whom this hospital ministers in Canning Town have more claim upon the giving public now than they have ever had.

**Central London Throat, Nose, and Ear Hospital, Gray's Inn Road.**—The work of this hospital has not been interrupted during the past or the present year. Eight beds were taken over by the War Office, and eighty-one sick and wounded soldiers have been treated during 1915. An annexe to the present building has been erected, giving accommodation for eighteen soldiers, who are sent by the military authorities for that treatment for which the hospital is specially equipped. The sick civil poor have not been deprived of the accommodation made for their benefit. Funds are diminished, and help is asked.

**Charing Cross Hospital.**—The chief need of this hospital at the present time is for increased contributions to keep pace with the ever-increasing cost of all the supplies which the hospital requires. The maintenance of the 300 beds costs at present £30,000 per annum, and although half these beds are occupied by military cases, for whom a grant of 4s. per head per day is received from the authorities, this does not cover the entire cost of their upkeep. Support is required not only to make good the deficit on the military side of the hospital, but also to provide the funds for carrying on the necessary work of looking after the civil population.

**Chelsea Hospital for Women, Fulham Road, S.W.**—It is estimated that the sum of about £30,000 is still required to enable the committee to complete the rebuilding fund. Special facilities are being given for the treatment of wives and other near relatives of soldiers at the Front. On building fund accounts £16,000 is now owing to the bankers (see also above), and the Council make a special appeal for contributions so that they may be able to report to Her Majesty that the financial outlook has much improved. The great increase in prices and dearth of legacies results in the general fund at the present moment being £1,500 overdrawn.

**Cheyne Hospital for Sick and Incurable Children, Chelsea.**—The special need of this hospital is money as, owing to the increased price of all stores and labour, the cost of working has greatly risen during the past year, and threatens to be higher still. At present the hospital depends entirely on legacies to meet the annual deficit. Balconies for the open-air treatment of tuberculous cases are greatly needed.

**Children's Hospital for the Treatment of Hip Disease, Sevenoaks.**—In answer to a special appeal, last year's deficit was cleared, but it is feared that, with the continued increase in prices, it will this year again be impossible to meet the extra cost without further public generosity. The hospital is now recognised under the Local Government Board as a hospital for the treatment of surgical tuberculosis for children under school age.

**City of London Hospital for Diseases of the Chest (Victoria Park, E.).**—This hospital, in connection with which is now established a tuberculosis dispensary treating patients from the Borough of Hackney, is amongst those which tend to suffer from the multifarious appeals in connection with the war. The work of the hospital has been sustained in full, but the conditions are becoming very difficult by reason of the serious increase in the cost of drugs and articles of diet. The hospital is especially in need of support at the present time owing to the unavoidable suspension of some of the subscriptions.

**City of London Lying-in Hospital.**—This hospital is still suffering from the effects of the heavy debt which was incurred when rebuilding in 1906. The rebuilding was absolutely essential on account of the construction of a tube railway opposite the hospital, which rendered the old structure unsafe, and it became necessary to borrow the sum of £24,000 from the bankers. After nine years, by the exercise of strict economy and persistent appeals, the loan last year was reduced to £4,700, but, owing to the high cost of food, drugs, etc., has had to be again increased to meet the ordinary current expenses. At the present time the work of a lying-in hospital is particularly important. This hospital welcomes without any formality the wives of our sailors and soldiers, 266 of whom received free treatment last year, besides a large number of Belgian and Russian refugees.

**East End Mothers' Lying-in Home, Commercial Road, E.**—To carry on the work of the home new subscriptions are wanted. The greater portion of the work is now being done among the wives of sailors and soldiers.

**East London Hospital for Children, Shadwell, E.**—Reduced incomes and money diverted to war funds have materially affected the funds of this hospital, which is at present unavoidably living on borrowed money, and, unless an unexpected windfall happens, will be obliged to continue to borrow until its security is exhausted. Many children are treated whose fathers are fighting at the Front, and who come to see them when on furlough. The board propose to hand over the convalescent home at Bognor for the use of the wounded.

**Epsom and Ewell Cottage Hospital, Surrey.**—In consequence of higher prices a large deficit for the

[*Continued on p.* 296.

### Nearly Two Million Sufferers Helped by our Hospitals.

### A SINGLE YEAR'S ROLL-CALL OF THE SICK.

IN the last year for which complete figures are available, the immense total of *one million nine hundred and fifty thousand seven hundred and twenty-eight* patients were treated at the voluntary hospitals and dispensaries of London, and the infectious hospitals of the Metropolitan Asylums Board. These figures include only the in-patient cases treated to a termination in the wards of the hospitals and the number of new out-patient cases treated in the out-patient departments and dispensaries, and may be taken as showing as nearly as possible the number of separate cases dealt with in the hospitals and dispensaries of the Metropolis. The total is approximately the same as in the previous year, being only about four thousand less, but in dividing the figure between the wards and the out-patient departments it is found that about thirteen thousand more in-patients were treated, and about seventeen thousand fewer out-patients. The extra beds for soldiers will partly account for the larger number of in-patients, and fewer attendances at the dispensaries for the fall in the number of out-patients.

852,276. Surgical Patients.

**Patients Suffering from Surgical Diseases.**—Of the whole number of patients received by the hospitals and dispensaries, *eight hundred and fifty-two thousand two hundred and seventy-six* required surgical treatment in addition to those treated in the special departments and in the hospitals for diseases of the eye, nose, throat, ear, etc. "Surgical" diseases include not only all accidents such as broken or fractured bones, cuts, burns, and all manner of displacements, crushings, and injuries of sensitive parts and organs, but also abscesses, ulcerations, cancers, and tumours of all kinds. Cases requiring Finsen light and Röntgen ray and similar treatment are classed under this heading, and the provision and upkeep of efficient curative apparatus make a steady call upon the resources of a hospital whose work includes such treatment. Over one million two hundred and twenty-four thousand patients are treated annually in the London hospitals for diseases requiring surgical treatment.

611,898. Medical Patients.

**Patients Suffering from Medical Diseases.** — *Six hundred and eleven thousand eight hundred and ninety-eight* persons received medical treatment. By medical diseases are meant those diseases which are situated either as to their source and origin or in their entirety in one or other of the three great cavities of the body. They include rheumatic fever, pneumonia, pleurisy, bronchitis, diseases of the stomach, bowels, liver, kidney, bladder, and pancreas, every kind of heart disease, many forms of brain injury, dyspepsia, constipation, the manifold diseases of the nervous system, and other ailments, many of them serious or dangerous to life, or at least to the useful existence of the individual. Unlike some surgical ailments with outward signs or symptoms, medical diseases are often out of sight; the diagnosis of their nature and extent, and the successful treatment of them, is dependent on the doctor's scientific knowledge. This knowledge is in the hospitals of London freely given to over seven hundred thousand patients by the foremost physicians of the day.

164,891. Eye.

**Patients Suffering from Eye Affections.**—*One hundred and sixty-four thousand eight hundred and ninety-one* persons were treated in the special departments of the general hospitals or by the ophthalmic hospitals of London. Special service was rendered to many anxious to fight their country's battles who required special treatment before passing the medical tests for enlistment. The blessing of unimpaired vision is appreciated by all, and the economic value of preserving and improving sight is incalculable. By this specialised treatment many patients have been saved from becoming practically helpless in the world.

### THE ROLL-CALL OF THE SICK.—*continued.*

181,114. Children.

**Patients Treated at Special Hospitals for Children.**—The total of patients mentioned at the commencement of this article include *one hundred and eighty-one thousand one hundred and fourteen* children sent from their own homes, where they could not be properly attended to, for treatment in the special hospitals for children. Of course, a great many more of our little ones were also treated at the general and other institutions. For obvious reasons the health of the rising generation is at the present time especially a valuable asset to the nation.

93,844.
Ear and Throat.

**Patients Suffering from Diseases of the Ear, Nose, and Throat.**—At the special hospitals or special departments devoted to these diseases *ninety-three thousand eight hundred and forty-four* patients were treated. The organs mentioned are intimately connected, and diseases and ailments affecting them involve temporary and often permanent impairment of the functions of hearing, swallowing, and breathing. Those in full health perhaps may find it difficult to understand, until experience has brought the fact home to them, what discomfort and loss of efficiency to the individual are caused by any affection of the ear, nose, or throat.

91,679. Women.

**Diseases of Women and Motherhood.**—*Ninety-one thousand six hundred and seventy-nine* women were treated at the Metropolitan voluntary hospitals for those diseases which are peculiar to their sex, or in the lying-in hospitals, where the best of our midwives are employed and trained. The very heart and strength of the nation lies in the home life, and the soul of the home life is the woman—the mother. Never was there a time when healthy and happy mothers were more valuable to the country than at the present day when motherhood, always sacred, is unusually precious.

55,564. Skin.

**Patients Suffering from Diseases of the Skin.**—During the year *fifty-five thousand five hundred and sixty-four* persons were treated for skin diseases in London. Although in these cases there is not, as a rule, the pain, or the danger to life, nor even such risk of permanent disablement as is the case with many other diseases, our sympathy is largely called for. The discomfort caused to the sufferer from these ailments and to his immediate friends is often considerable, and the expense of adequate treatment is so great that it is difficult to realise what the result would be were there no hospitals for the relief of this kind of patient.

36,832. Fever.

**Patients Suffering from Fever.**—The number of patients treated was *thirty-six thousand eight hundred and thirty-two.* The diseases here classified include scarlet fever, diphtheria, and measles; the latter has prevailed to such an extent in London during recent years that more deaths have occurred from it than from scarlet fever. Many families have experienced the emergency when the value of the Fever Hospital is appreciated.

29,219.
Consumption.

**Patients Suffering from Consumption.**—*Twenty-nine thousand two hundred and nineteen* patients suffering from phthisis or consumption, or diseases of the chest, were treated in the hospitals of London during the year. Most of us have seen something of the ravages and cruelty of consumption, and all dread this terrible disease, which may be called the curse of our climate. By it neither persons nor estate, rich nor poor, old nor young, are respected.

14,525.
Paralysis.

**Patients Suffering from Paralysis, Epilepsy, and Nervous Diseases.**—*Fourteen thousand five hundred and twenty-five* persons stricken by paralysis, epilepsy, neuritis, neuralgia, neurasthenia and kindred ailments received treatment at the general hospitals and hospitals devoted to these maladies. The hurry and stress of modern life reap a terrible harvest, especially in a vast centre like London, where it is impossible to dissociate nervous breakdown from the toil and hurry of existence. No disease is more sudden than paralysis, surely none claiming more pity for its victims, often struck down without the slightest warning.

This is the story and roll-call of an army of sufferers, numbering nearly two millions, who have received treatment over an extended period within the year under review. Again they claim our sympathy and help. To the vigorous, to those in health who are able to provide for their dependants, to those who know what ill-health means, who have suffered from disease of one kind or another, and who, either in the hospital or under the skill and care of the doctors and nurses trained in the hospitals, have been restored to health and usefulness, we confidently appeal on behalf of the London hospitals.

### THE ROLL-CALL OF THE SICK.

| | | | |
|---|---|---|---|
| Sufferers needing Surgical Aid . . . . | 852,276 | Sufferers from Skin Diseases. . . . | 55,564 |
| Sufferers needing Medical Care . . . . | 611,898 | Fever Patients . . . . . . . . | 36,832 |
| Sufferers from Eye Troubles . . . . | 164,891 | Consumptives . . . . . . . | 29,219 |
| Diseases of the Ear, Nose, and Throat . | 93,844 | Paralysis and Epilepsy . . . . . | 14,525 |
| Diseases of Women . . . . | 91,679 | Total . . . . | 1,950,728 |

### Hospitals and their Special Needs.

[Continued from p. 293.

current year is expected. Cases from the Public Schools Battalions of the Royal Fusiliers quartered at Epsom have been treated.

**Evelina Hospital for Children, Southwark Bridge Road, S.E.**—Owing to its situation, the "Evelina" is rarely seen by wealthy people, and suffers accordingly, although it is the only large children's hospital for the whole of South London, and is situated in its most destitute district. Although obviously nothing can be done for the wounded at the "Evelina," practically all of its patients are the little ones of our sailors and soldiers. The Evelina Hospital is doing exceedingly good work, as may be gathered from the fact that over 1,000 in-patients and about 50,000 out-patients are dealt with each year.

**Eversfield Chest Hospital, West Hill Road, St. Leonards-on-Sea.**—Annual subscriptions and donations are needed to maintain the hospital in its present state of efficiency. A sum of £1,250 is required to carry to completion and to pay off the debt in connection with the finished section of the cliff wall that it has been necessary to erect to safeguard the hospital buildings which were being menaced by the serious crumbling of the cliff bounding the grounds on the south side. A further sum of £4,500 is also needed to pay off the mortgage.

**French Hospital and Dispensary, Shaftesbury Avenue.**—This hospital is open to all foreigners speaking French, and in 1915 735 patients were admitted. The hospital placed thirty beds at the disposal of the British War Office and Admiralty, and the French Convalescent Home at Brighton also offered forty-six beds. Over 600 wounded (mostly English) have been received, and ten Belgian refugees were also given shelter at the convalescent home.

**Great Northern Central Hospital, Holloway Road.** This, the largest general hospital in North London, with 284 beds in London and Clacton, treats 2,900 in-patients annually, and there are 91,000 out-patient attendances. The cost hitherto has been about £21,000 a year, the greater part of which has to be obtained from voluntary sources. The committee have placed 150 beds at the disposal of the military authorities for sick and wounded soldiers, and already a thousand wounded have passed through the wards. For this purpose a large number of additional beds have been provided, entailing additional expenditure. No less than from £25,000 to £26,000 will be required in this the Diamond Jubilee year of the hospital's existence. The original scheme for a nurses' home has been elaborated, so as to include an institute of private nurses, and the total cost is estimated at £25,000. Of this £2,000 has now been collected.

**Grosvenor Hospital for Women, Vincent Square, S.W.**— Starting the year with a deficit of nearly £300, the reduction of income and the increasing price of all necessaries continuing, the hospital is in special need of funds at this time. The military authorities have been allotted 10 beds for sick nurses, and these are kept constantly occupied. A greater number of beds for the purpose has been asked for, but the needs of our ordinary patients preclude the provision of them. Contributions will be gladly received by the Secretary, Mr. W. J. Davidson.

**Guy's Hospital, S.E.**—The work of this hospital has been fully and efficiently maintained during the present time of strain. Economy, however, both of time and money, is now more than ever to be desired, and the friends of Guy's Hospital will realise fully that in its primary aim—the comfort and healing of the sick—but little economy is possible, and that the work of the institution must be maintained at whatever cost. In addition to the treatment of the sick poor of the civil population, the governors have set aside a special ward of forty-six cubicles for the reception of wounded officers, and a considerable number of men have received treatment for minor surgical ailments to enable them to enter upon active service, a further ward having been set apart for this purpose. The governors are glad to think that, in these difficult times, there has been no slackening of this beneficent work, and they are confident that the heavy expenditure inevitably incurred, exceeding the income in the past year by a considerable sum, will be a stimulus to the generosity of those who have the welfare of the hospital at heart. In 1915, 110,932 out-patients and 9,900 in-patients were treated. The ordinary expenditure amounted to £82,307, and the income from endowments to £45,320. The governors earnestly appeal for new annual subscriptions and donations to provide for the large deficiency between assured income and ordinary outgoings, which, in 1915, amounted to £36,987, and for which the hospital is entirely dependent upon voluntary support.

**Hampstead General and North-West London Hospital.**—The amalgamation of these two hospitals was effected in 1908 through the instrumentality of King Edward's Hospital Fund, and 1,439 patients were admitted to the hospital during the past year, while the attendances of the out-patients' department averaged over 1,000 weekly. But the development of the hospital in the special circumstances indicated has been more rapid than the expansion of its income, and the total indebtedness of the institution is now £5,000. The Council consequently make an earnest appeal for new annual subscriptions and donations. Over 350 wounded soldiers have been treated, and fifty-three beds are held at the disposal of the War Office.

**Hospital and Home for Incurable Children, Hampstead, N.W.**—The special need of this hospital at present is money for the ordinary maintenance, upkeep, and work of the institution. There is an overdraft of £125 at the bank.

**Hospital for Consumption, Brompton, S.W.**—For seventy-five years this has been the leading London hospital for chest troubles. Including the Sanatorium at Frimley it has about 500 beds constantly occupied. Many British sailors and soldiers, as well as French and Belgian soldiers, have been admitted to the Brompton Hospital, and also to the Sanatorium and Convalescent Home at Frimley since the outbreak of the war. Special arrangements have also been made for the admission of dependants of sailors and soldiers serving with H.M. Forces if needing treatment. Money is the one essential required, and the committee appeal to the generous and patriotic public to come to their aid with funds sufficient to carry on the work.

**Hospital for Sick Children, Great Ormond Street, W.C.**—It is anticipated that, unless the unexpected happens during the year 1916, a deficit between assured income and expenditure of about £2,000 will have to be met to keep the hospital out of debt. The need for greater effort to counterbalance the drain of war upon the manhood of the nation, by saving infant life for the future welfare of the British Empire, compels the committee to plead most earnestly for continued and increased support for the national work this hospital is performing in the preservation of child-life.

**Hospital for Women, Soho Square, W.**—During 1914 1,056 patients were admitted, and 14,119 patients attended in the out-patient department, while there were usually 100 patients or more on the waiting list for admission. New annual subscribers are wanted to help to reduce the mortgage debt, which now stands at £4,500, and costs the hospital £202 10s. a year for interest alone.

**Infants Hospital, Vincent Square, Westminster, S.W.**—This hospital is at the present time £1,200 in debt on account of expenses of maintenance, and, after reckoning all the money in prospect—chiefly promised subscriptions—there remains a further sum of about £1,750 to be raised to meet the expense of maintenance for this year. Besides carrying on the usual work of the hospital, which is now, if possible, more important than ever, the Committee has offered accommodation to the War Office, although the offer has not yet been accepted.

**King's College Hospital, Denmark Hill, S.E.**—The removal of the hospital from the Strand district to South London has unfortunately had a serious effect on its finances. It has necessarily lost its local connection, and is only gradually building up a new one in South London. It is hoped, however, that residents in this district will liberally respond, for the hospital is in urgent need of support for its ordinary work, and is still heavily burdened by a large debt on the new buildings, which have now all been taken into use. On the outbreak of the war a considerable portion of the hospital was placed at the disposal of the War Office for the use of the 4th London General Hospital, retaining the casualty department, and at least four wards for the use of the civilian population. The buildings are well adapted for the purpose, and it has been found possible to accommodate upwards of 700 patients, together with the increased staff which these numbers entail. It is urgently hoped that the work may not be crippled in any way through lack of funds. An appeal is being made for £100,000 to pay off the debt on the building fund, and to provide for present and future requirements.

**London Homœopathic Hospital, Great Ormond Street, W.C.**—To provide the sum to be raised by December 31 of the current year to pay off debts to capital, £14,504, unavoidably incurred in the maintenance of the hospital, many generous donations are necessary, and it is hoped that substantial help will be forthcoming before the year closes.

**London Hospital, Whitechapel, E.**—The work of the London Hospital continues to increase, and during the past year a greater number of patients have been treated there than in any previous year. Despite the requirements of the war, by slightly rearranging the wards the number of beds available for civilians has not been seriously curtailed. By reducing research work and limiting the functions of the hospital to the actual treatment of patients, many doctors and nurses have been enabled to join the Forces. At the same time, while all schemes involving outlay of capital have as far as possible been postponed and the hospital has retrenched its expenditure in various ways, funds are urgently needed to carry on the work. In 1915 18,933 ordinary in-patients were treated alone, and since the war there has been a general rise in the price of all commodities: drugs and chemicals were, in particular, affected; certain of German origin were unobtainable. Voluntary contributions, moreover, were being diverted from the hospital to the support of charities of a more directly patriotic nature. In addition to this, the London Hospital placed 500 beds at the disposal of the naval and military authorities. Up to December 31, 1915, 3,277 wounded had been treated. To meet the demand for nurses a large number of paying probationers have been received, and great numbers of orderlies have been trained.

**London Lock Hospitals, Harrow Road, W., and Dean Street, W.C.**—The importance of the work that these hospitals carry on is best demonstrated by a perusal of the final report of the Royal Commission on Venereal Diseases, March 1916, where it is stated that "the disastrous effects of congenital syphilis can be obviated by efficient treatment of the parents of the children, or both." This policy has been in force at the London Lock Hospitals for years. The committee have just issued an emergency appeal for £3,500, in order to pay tradesmen's accounts owing by June 30. The male hospital is busy treating both English and Belgian soldiers, who are enabled, through the relief afforded and advice given, to return to the firing line cured. All the children and a great many of the other patients are suffering through no fault of their own, and the wards at this hospital contain some of the most pathetic cases in the kingdom.

**London Temperance Hospital.**—This hospital was founded in 1873 to demonstrate the possibility of successfully treating disease and personal injuries without the (at that time) universal use of alcohol. It is a general public hospital, situate in Hampstead Road, and is thoroughly equipped and up to date, having, in addition to the usual medical and surgical departments, ophthalmic, skin, ear, nose, and throat and dental departments. There are 120 available beds, and out-patient and casualty departments. At the outbreak of the war thirty beds were placed at the disposal of the War Office, and during the past year 200 soldiers, sick or wounded, were received as in-patients. The board feel confident that a larger knowledge of the work done there will result in much greater financial support to meet the increase of cost of maintenance. The expenses are over £10,000 per annum, while the donations and subscriptions are about £2,000 per annum.

**Metropolitan Ear, Nose, and Throat Hospital, Fitzroy Square, W.**—When the war broke out this hospital was placed at the disposal of the naval and military authorities. Beds were set apart, and have

[*Continued on p.* 300.

### 1915.

### A YEAR'S WORK IN THE HOSPITALS AND MEDICAL CHARITIES OF LONDON.

### ST. MARYLEBONE AND WEST CENTRAL DISTRICT.

Comprising St. Marylebone, St. John's Wood, Bloomsbury, Holborn, &c.

| No. of Beds. | No. of Beds Daily Occupied. | HOSPITALS. | In-patients. | Out-patient Attendances. | Total Expenditure. | Income. Charitable. | Income. Proprietary. | Income. Patients' Payments. | Total Income. | Legacies not included in preceding column. |
|---|---|---|---|---|---|---|---|---|---|---|
| | | | | | £ | £ | £ | £ | £ | £ |
| 74 | 51 | French ... ... ... ... | 737 | 18,992 | 6,136 | 5,121 | 1,839 | 1,207 | 8,167 | 3,262 |
| 80 | 46 | Italian ... ... ... ... | 751 | 8,257 | 4,162 | 1,970 | 1,708 | 475 | 4,153 | 777 |
| 164 | 108 | London Homœopathic ... ... | 1,504 | 55,323 | 15,488 | 5,093 | 3,814 | 2,660 | 11,567 | 610 |
| 151 | 135 | SS. John and Elizabeth ... ... | 1,314 | ... | 12,088 | 1,951 | 7,916 | 2,041 | 11,908 | 380 |
| 482 | 357 | The Middlesex ... ... ... | 7,611 | 155,211 | 48,995 | 16,424 | 12,207 | ... | 28,631 | 16,270 |
| 90 | 88 | Alexandra, for Children... ... | 118 | 1,000 | 5,344 | 3,428 | 1,138 | 1,916 | 6,482 | 5 |
| 250 | 234 | Hospital for Sick Children ... | 2,958 | 84,769 | 25,614 | 10,451 | 6,334 | 1,393 | 18,178 | 3,894 |
| 24 | 22 | S. Monica's, for Children ... | 156 | ... | 1,409 | 649 | 237 | 297 | 1,183 | 250 |
| 82 | 71 | Queen Charlotte's Lying-In ... | 1,926 | 22,230 | 8,021 | 5,003 | 927 | 854 | 6,784 | ... |
| 71 | 67 | New Hospital for Women ... | 1,308 | 34,486 | 8,363 | 3,448 | 1,248 | 2,323 | 7,019 | 5,075 |
| 67 | 52 | Samaritan Free ... ... ... | 1,015 | 12,300 | 6,709 | 4,689 | 489 | ... | 5,178 | 814 |
| 207 | 181 | National for the Paralysed, &c.... | 1,064 | 48,044 | 23,798 | 9,505 | 3,003 | 6,990 | 19,498 | 4,876 |
| 70 | 70 | Hospital for Epilepsy, &c. ... | 450 | 22,871 | 6,411 | 4,235 | 234 | 2,211 | 6,680 | 1,135 |
| 72 | 66 | West End, for Epilepsy, &c. ... | 305 | 34,230 | 8,085 | 4,098 | 1,927 | 2,190 | 8,215 | 520 |
| 28 | 18 | Central London Ophthalmic ... | 355 | 29,206 | 2,685 | 3,206 | 56 | ... | 3,262 | 600 |
| 16 | 15 | Western Ophthalmic ... ... | 393 | 26,020 | 2,144 | 1,943 | 111 | 420 | 2,474 | 100 |
| 183 | 136 | Royal National Orthopædic ... | 1,434 | 31,368 | 14,864 | 7,857 | 1,713 | 6,638 | 16,208 | 479 |
| 38 | 30 | Florence Nightingale Home ... | 469 | ... | 4,716 | 2,240 | 283 | 2,724 | 5,247 | 500 |
| ... | ... | National Dental ... ... ... | ... | 22,087 | 2,120 | 742 | 5 | 1,042 | 1,789 | ... |
| 14 | 8 | London Throat ... ... ... | 406 | 9,425 | 1,281 | 667 | 2 | 745 | 1,414 | ... |
| 49 | 46 | The Middlesex Cancer ... ... | 176 | 724 | 5,760 | 2,399 | 2,573 | ... | 4,972 | 418 |
| 20 | 16 | Metropolitan Ear, &c. ... ... | 501 | 11,172 | •2,070 | 739 | 48 | 1,292 | 2,079 | ... |
| 2,232 | 1,817 | | 24,951 | 627,715 | 216,263 | 95,858 | 47,812 | 37,418 | 181,088 | 39,965 |
| | | DISPENSARIES. | | | | | | | | |
| ... | ... | London Medical Mission ... | ... | 21,533 | 1,933 | 597 | 416 | 272 | 1,285 | ... |
| ... | ... | Margaret Street, for Consumption | ... | 7,561 | 812 | 471 | 21 | ... | 492 | 2,353 |
| ... | ... | St. John's Wood Provident ... | ... | 11,300 | 508 | 256 | 26 | 182 | 464 | 10 |
| ... | ... | St. Marylebone General... ... | ... | 16,358 | 1,225 | 647 | 186 | 236 | 1,069 | ... |
| ... | ... | Western General... ... ... | ... | 19,442 | 891 | 610 | 143 | 26 | 779 | ... |
| 2,232 | 1,817 | | 24,951 | 703,909 | 221,632 | 98,439 | 48,604 | 38,134 | 185,177 | 42,328 |

### WESTMINSTER DISTRICT.—Comprising Westminster City and Liberties.

| No. of Beds. | No. of Beds Daily Occupied. | HOSPITALS. | In-patients. | Out-patient Attendances. | Total Expenditure. | Income. Charitable. | Income. Proprietary. | Income. Patients' Payments. | Total Income. | Legacies not included in preceding column. |
|---|---|---|---|---|---|---|---|---|---|---|
| | | | | | £ | £ | £ | £ | £ | £ |
| 300 | 205 | Charing Cross ... ... ... | 2,989 | 75,875 | 30,913 | 35,375 | 4,944 | 1,768 | 42,087 | 50 |
| 213 | 179 | Westminster ... ... ... | 2,045 | 57,098 | 24,219 | 12,087 | 4,881 | 5,465 | 22,433 | 1,856 |
| 162 | 156 | Ventnor, for Consumption ... | 497 | ... | 15,458 | 4,810 | 3,511 | 7,546 | 15,867 | 2,100 |
| 37 | 25 | Grosvenor, for Women & Children | 367 | 5,798 | 2,935 | 1,409 | 464 | 691 | 2,564 | 50 |
| 67 | 66 | Hospital for Women ... ... | 1,056 | 14,119 | 7,016 | 4,140 | 665 | 1,263 | 6,068 | 578 |
| 25 | 19 | Gordon, for Fistula ... ... | 311 | 2,995 | 2,397 | 577 | ... | 1,782 | 2,359 | 20 |
| 24 | 24 | National, for Diseases of Heart... | 181 | 15,049 | 3,629 | 2,028 | 379 | 678 | 3,085 | 2,067 |
| 40 | 30 | Royal Westminster Ophthalmic... | 790 | 38,359 | 3,875 | 2,104 | 1,324 | 83 | 3,511 | 1,260 |
| 20 | 1 | Royal Ear ... ... ... ... | 37 | 555 | 922 | 789 | 36 | 46 | 871 | 750 |
| ... | ... | Royal Dental ... ... ... | ... | 70,855 | 6,351 | 6,500 | 1,184 | 1,737 | 9,421 | ... |
| 32 | 29 | St. Peter's, for Stone ... ... | 486 | 35,511 | 4,849 | 1,779 | 440 | 2,489 | 4,708 | 25 |
| 50 | 33 | Infants' Hospital, Vincent Square | 343 | 3,560 | 3,329 | 2,781 | 76 | ... | 2,857 | ... |
| 40 | 33 | St. John's, for Skin Diseases ... | 219 | 35,735 | 4,360 | 2,250 | 66 | 2,106 | 4,422 | ... |
| 57 | 25 | Throat Hospital, Golden Square | 789 | 47,383 | 6,735 | 2,073 | 61 | 4,485 | 6,619 | ... |
| 1,067 | 825 | | 10,110 | 402,892 | 116,988 | 78,702 | 18,031 | 30,139 | 126,872 | 8,756 |
| | | DISPENSARIES. | | | | | | | | |
| ... | ... | Public ... ... ... ... ... | ... | 9,304 | 750 | 304 | 214 | ... | 518 | 150 |
| ... | ... | St. George's, Hanover Square ... | ... | 1,541 | 401 | 261 | 54 | 93 | 408 | ... |
| ... | ... | Western ... ... ... ... | ... | 12,632 | 1,322 | 285 | 477 | 634 | 1,396 | ... |
| ... | ... | Westminster General ... ... | ... | 11,565 | 724 | 360 | 301 | 88 | 749 | ... |
| 1,067 | 825 | | 10,110 | 437,934 | 120,185 | 79,912 | 19,077 | 30,954 | 129,943 | 8,906 |

### CITY AND EAST CENTRAL DISTRICT.

Comprising the City, St. Luke's, Shoreditch, Finsbury, and Clerkenwell.

| No. of Beds. | No. of Beds Daily Occupied. | Hospitals. | In-patients. | Out-patient Attendances. | Total Expenditure. | Income. | | | Total Income. | Legacies not included in preceding column. |
|---|---|---|---|---|---|---|---|---|---|---|
| | | | | | | Charitable. | Proprietary. | Patients' Payments. | | |
| | | | | | £ | £ | £ | £ | £ | £ |
| 360 | 145 | Metropolitan ... ... ... | 1,979 | 130,680 | 19,331 | 11,931 | 1,160 | 3,325 | 16,416 | 280 |
| 175 | 153 | Royal Free ... ... ... | 2,346 | 115,375 | 23,966 | 9,411 | 4,193 | 1,779 | 15,383 | 8,211 |
| 689 | 581 | St. Bartholomew's ... ... | 8,648 | 261,864 | 99,688 | 24,257 | 75,693 | 12,537 | 112,487 | 1,587 |
| 80 | 56 | Royal, for Diseases of the Chest. | 370 | 17,774 | 8,100 | 4,051 | 937 | 1,897 | 6,885 | 148 |
| 164 | 163 | Queen's, for Children ... ... | 2,029 | 98,346 | 17,955 | 13,318 | 829 | 552 | 14,699 | 1,108 |
| 61 | 46 | City of London Lying-In ... | 1,100 | 8,225 | 6,818 | 1,734 | 3,701 | 675 | 6,110 | 500 |
| 52 | 42 | St. Mark's, for Fistula ... ... | 737 | 6,166 | 6,025 | 4,231 | 1,354 | 6 | 5,591 | 553 |
| 138 | 111 | Royal London Ophthalmic ... | 2,208 | 123,783 | 15,059 | 10,120 | 2,095 | 699 | 12,914 | 1,742 |
| 30 | 26 | Central London Throat and Ear. | 677 | 45,002 | 4,382 | 1,549 | 362 | 3,360 | 5,271 | 1,009 |
| 1,749 | 1,323 | | 20,094 | 807,215 | 201,324 | 80,602 | 90,324 | 24,830 | 195,756 | 15,138 |
| | | DISPENSARIES. | | | | | | | | |
| ... | ... | Billingsgate Medical Mission ... | ... | 9,346 | 645 | 545 | ... | 57 | 602 | ... |
| ... | ... | City ... ... ... ... | ... | 15,793 | 911 | 826 | 84 | ... | 910 | ... |
| ... | ... | Farringdon General ... ... | ... | 9,273 | 543 | 347 | 31 | 185 | 563 | ... |
| ... | ... | Finsbury ... ... ... ... | ... | 24,013 | 925 | 416 | 165 | 211 | 792 | ... |
| ... | ... | Metropolitan ... ... ... | ... | 10,017 | 631 | 214 | 259 | 84 | 557 | ... |
| ... | ... | Royal General ... ... ... | ... | 6,584 | 925 | 194 | 401 | 55 | 650 | ... |
| 1,749 | 1,323 | | 20,094 | 882,241 | 205,904 | 83,144 | 91,264 | 25,422 | 199,830 | 15,138 |

### ISLINGTON AND NORTH-WEST DISTRICT.

Comprising Islington, Holloway, Highbury, Hampstead, Highgate, St. Pancras, Stoke Newington, Tottenham, &c.

| No. of Beds. | No. of Beds Daily Occupied. | Hospitals. | In-patients. | Out-patient Attendances. | Total Expenditure. | Income. | | | Total Income. | Legacies not included in preceding column. |
|---|---|---|---|---|---|---|---|---|---|---|
| | | | | | | Charitable. | Proprietary. | Patients' Payments. | | |
| | | | | | £ | £ | £ | £ | £ | £ |
| 224 | 205 | Great Northern Central ... ... | 2,707 | 90,135 | 26,058 | 14,972 | 7,980 | 1,204 | 24,156 | 2,388 |
| 141 | 105 | Hampstead General Hospital ... | 1,232 | 45,812 | 13,266 | 8,278 | 639 | 3,354 | 12,271 | 5,127 |
| 116 | 92 | London Temperance ... ... | 1,239 | 75,710 | 10,432 | 4,533 | 2,107 | 901 | 7,541 | 150 |
| 125 | 111 | Tottenham (Prince of Wales's) .. | 1,561 | 78,694 | 12,454 | 12,002 | 302 | 22 | 12,326 | 67 |
| 350 | 324 | University College ... ... | 4,643 | 169,645 | 33,268 | 17,531 | 5,693 | 9,464 | 32,688 | 6,812 |
| 110 | 102 | Mount Vernon, for Consumption | 402 | 12,950 | 11,351 | 2,955 | 2,481 | 5,126 | 10,562 | 847 |
| 20 | 20 | Children's Home Hospital, Barnet | 28 | ... | 634 | 420 | 228 | 81 | 729 | ... |
| 160 | 72 | London Fever ... ... ... | 949 | ... | 12,676 | 6,004 | 1,684 | 3,684 | 11,372 | 220 |
| 26 | 16 | Invalid Asylum ... ... ... | 163 | ... | 1,292 | 360 | 370 | 219 | 949 | ... |
| 22 | 6 | Bushey Heath Cottage ... ... | 85 | ... | 619 | 325 | 100 | 114 | 539 | 50 |
| 25 | 19 | Enfield Cottage ... ... ... | 252 | ... | 1,353 | 1,063 | 231 | 172 | 1,466 | 45 |
| 25 | 12 | St. Saviour's Hospital ... ... | 153 | ... | 2,087 | 930 | 156 | 897 | 1,983 | 3,421 |
| 48 | 40 | St. Columba's Hospital ... ... | 128 | ... | 3,855 | 1,935 | 429 | 450 | 2,814 | 25 |
| 58 | 38 | Willesden Cottage ... ... | 553 | 588 | 2,115 | 1,516 | 206 | 262 | 1,984 | ... |
| 30 | 19 | Wood Green Cottage ... ... | 279 | 73 | 1,621 | 1,052 | 74 | 539 | 1,665 | ... |
| 20 | 15 | Santa Claus Home ... ... | 45 | ... | 955 | 786 | 39 | 30 | 855 | ... |
| 56 | 55 | Hospital for Incurable Children | 29 | ... | 2,271 | 622 | 568 | 1,006 | 2,196 | 20 |
| 18 | 17 | Winifred House, Holloway ... | 14 | ... | 817 | 484 | 74 | 186 | 744 | 37 |
| 18 | 16 | Highgate Convalescent Home ... | 140 | ... | 497 | 495 | 21 | 4 | 520 | ... |
| 1,592 | 1,284 | | 14,602 | 473,607 | 137,621 | 76,263 | 23,382 | 27,715 | 127,360 | 19,209 |
| | | DISPENSARIES | | | | | | | | |
| ... | ... | Child's Hill Provident ... ... | ... | 2,770 | 103 | 18 | 10 | 74 | 102 | ... |
| ... | ... | Hampstead Provident ... ... | ... | 10,884 | 810 | 251 | 62 | 533 | 846 | ... |
| ... | ... | Islington ... ... ... ... | ... | 43,755 | 774 | 212 | 34 | 544 | 790 | ... |
| ... | ... | Islington Medical Mission ... | ... | 9,611 | 691 | 483 | 187 | 57 | 727 | ... |
| ... | ... | Kentish Town Medical Mission... | ... | 2,769 | 167 | 139 | 6 | 8 | 153 | ... |
| ... | ... | St. Pancras and Northern ... | ... | 10,490 | 973 | 344 | 134 | 481 | 959 | ... |
| ... | ... | Stamford Hill, &c. ... ... | ... | 18,547 | 640 | 461 | 159 | ... | 620 | ... |
| 1,592 | 1,284 | | 14,602 | 572,433 | 141,779 | 78,171 | 23,974 | 29,412 | 131,557 | 19,209 |

[*For remaining tables see pages* 302 *and* 303.

### Hospitals and their Special Need

[Continued from p. 297.

been continuously occupied by the wounded, without precluding at the same time adequate treatment of civilian patients. In addition to the wounded in the wards, a large number of soldiers have been treated in the out-patient department, not only from the Front, but also from amongst those under training to go there. The work occasioned by the war has been a great strain on the finances of the hospital, and donations and subscriptions are urgently needed.

**Metropolitan Hospital, Kingsland Road, N.E.**—The most recent events in the war have confirmed the committee in the wisdom of making ample provision for the civilian population during its progress, having regard to the circumstances of the district and its population. Attached to the hospital there was opened, in October 1915, the adjacent L.C.C. Enfield Road School, for the accommodation of 202 sick and wounded soldiers. It is urgently hoped that all friends and supporters of this excellent and thoroughly deserving charity will not only lend their personal aid, but will urge their friends to do so. For those who cannot send money, gifts of clothing, food, fruit, eggs, etc., and flowers are very acceptable.

**Middlesex Hospital, W.**—The governors ask for the help the hospital needs to enable it to meet its heavy responsibilities. While caring for civilian patients as in normal times, the hospital has, since the outbreak of the war, successfully treated 4,004 wounded members of the Expeditionary Force in London and at the branch hospital at Clacton-on-Sea, a worthy record of service which should appeal most strongly to those who, debarred from active service themselves, are anxious and able to do their part to bring the common task to successful fulfilment. Important as the nursing back to health of wounded men may be, the maintenance of the health of the civil portion of the community has its value, and the results of the hospital's work in that connection could be expressed in terms of industrial efficiency, a factor as essential to ultimate success as the equipment of an army for the field. The Cancer Charity, which, during the last century and a quarter, has done an amount of good for suffering humanity that is simply incalculable, is attached to the hospital. The accounts for last year show a heavy deficit, and an appeal has been made to the public asking for financial assistance to provide for the continuance of the Charity's work.

**Mildmay Mission Hospital.**—Owing to serious defects the entire drainage system of this hospital has to be reconstructed. The cost of this work will be over £1,700, and at present the hospital has no funds available.

**Miller General Hospital for S.E. London, Greenwich Road, S.E.**—Contributions are needed towards making good the deficiency in income which will be caused in this year's accounts by the war. The work of the hospital has been carried on, but not without difficulty, and already this year the expenditure has exceeded the income by £834. A debt of £4,400 still remains unpaid on the new wing, and a scheme for providing new out-patients' buildings necessarily remains in abeyance. A ward has been set apart for wounded, and this has been continuously occupied. The hospital needs of the civilian population have not been lost sight of, and many more beds have been occupied by them than before the war.

**Mount Vernon Hospital for Consumption and Diseases of the Chest, Northwood.**—The committee are in great need of additional help. They provide for those who are excluded from the benefits of the Insurance Act, and special provision is made for throat and heart cases and for children. The hospital is delightfully situated amid charming surroundings, in the fresh, pure air of the country, which has not been inaptly described as "Switzerland in Middlesex." Funds are urgently needed to continue the good work and to avoid debt. In view of the demand for admission any curtailment is not to be thought of, but the increased cost of all articles makes great inroads upon the available income, which needs substantially to be augmented.

**National Dental Hospital (Dental Department of University College Hospital), Great Portland Street.**—Since the war broke out this special hospital has treated over six thousand soldiers free, for gas extractions and fillings, besides providing over one hundred of them with free dentures. The numbers of ordinary patients have increased, and among these there is a continuous increase in the number who have to be treated quite free, and a decrease in the number who can afford to pay the very small charges asked from those who are unable to pay a dentist's charges.

**National Hospital for Paralysed and Epileptic, Queen Square, W.C.**—An appeal is made for new annual subscriptions and donations to meet the increased cost of maintenance which in many ways has particularly affected this institution, which has to purchase expensive drugs, etc., on a large scale. There are seventy beds for soldiers suffering from nerve injuries and severe mental and nervous shock. Every effort is being made to provide accommodation for the needs of the civil public, and extra beds have been temporarily added.

**North London or University College Hospital, W.C.** In order to provide additional beds to meet military requirements, a temporary annexe hospital, with accommodation for over 100 soldiers, has been established in the new eugenics laboratory of University College, kindly placed at the disposal of the hospital for this special purpose by the college authorities. The total number of beds has thus been temporarily increased to 447, 210 of which are allotted to sailors and soldiers. The total number of in-patients treated in the wards was 4,643, of whom over 1,000 were wounded sailors and soldiers. The total number of out-patients treated in the various departments was 58,896. New features of the hospital are the dental department in Great Portland Street (formerly the National Dental Hospital, recently amalgamated with University College Hospital), the maternity and child welfare department, and the tuberculosis dispensary. The hospital is almost entirely dependent on voluntary contributions.

**Paddington Green Children's Hospital, W.**—The hospital provides forty-six beds, and there is accommodation at the Convalescent Home, "Fair View," Slough, for sixteen children in the winter and twenty-four in the summer months. The average yearly number of hospital in-patients is over 700, of new out-patients 16,000, and of out-patient attendances 50,000. Over 40 per cent. of the in-patients are sailors' and soldiers' children, while a large number of such children are treated in the out-patient department. The total expenditure of the hospital during 1915, including a transfer of £500 to the Convalescent Home, amounted to £5,705, and the ordinary income to £4,328, a deficiency of £1,377. The amount owing by the hospital to its bankers on December 31, 1915, was £1,000, and this has since been increased during 1916 by further amounts, borrowed to meet necessary current expenses, to £1,920. Sir Douglas Owen, Chairman; Nigel Hanbury, Esq., Treasurer; W. H. Pearce, Secretary.

**Passmore Edwards Hospital for Willesden, Harlesden Road, N.W.**—There are now 60 beds instead of twenty-five, and funds are much wanted to enable this hospital to treat its soldier and civilian patients and to pay for an *x*-ray department recently erected.

**Poplar Hospital for Accidents, East India Dock Road, Poplar, E.**—Funds are needed to enable the hospital to continue to pay its way. Patients are treated at the rate of six per hour, day and night, and for sixty years the hospital has been free from debt, but the great increase in food, drugs, and dressings, and increased salaries, will make our task very much harder. Twenty-four beds have been set aside for wounded soldiers, but only twelve have been withdrawn from our civilian patients.

**Queen Charlotte's Lying-in Hospital, Marylebone Road, N.W.**—The hospital is in great need of funds for maintenance. Owing to the falling off in subscriptions and donations and to the considerable increase in the price of food and practically all other requirements the hospital is in debt to the extent of £5,000. Since the outbreak of war nearly 2,000 wives of our sailors and soldiers have been admitted to the wards or attended and nursed at home, as well as many Belgian and other refugees. Further contributions are earnestly solicited.

**Queen's Hospital for Children, Bethnal Green, E.**—The demands upon this hospital continue to grow, last year's figures showing that 42,225 cases (making 98,346 attendances) passed through the out-patient department, while 2,029 children were admitted to the wards in London and 212 to those at the Bexhill branch—"The Little Folks' Home." The cost of this work under present conditions is, roughly, £18,000 a year, towards which only £500 is obtained from endowments. The expenditure of 1915 shows an increase of £716 over that of 1914, which works out at 4½ per cent. on the previous year's cost. The average prices of most supplies were at least 20 per cent. higher than in 1914, and it is therefore evident that war-time economies have been properly adopted. In co-operation with other charities and with the local medical officers of health, the hospital carries on every summer an extensive propaganda against summer diarrhœa, which comes as a dreaded scourge with the hot, dry weather year after year.

**Royal Ear Hospital, Dean Street, Soho, W.**—For a century this hospital has been the means of conferring great benefits to the sick poor suffering from deafness and all diseases of the ear. It is unendowed, and a building debt of £3,000 hangs like a millstone round its neck. Beds were gratuitously placed at the disposal of the Admiralty for naval men suffering from deafness or ear injuries. It is hoped that the numerous friends of the hospital, bearing these facts in mind, will give it increased support this, its centenary, year.

**Royal Free Hospital, Gray's Inn Road, W.C.**—2,504 patients were admitted to the wards and 115,375 attendances of out-patients are recorded for the past year. On behalf of the Country's Charge Fund for further providing for infant welfare an appeal for £200,000 is made. This will enable the hospital authorities to provide ante-natal and infant welfare wards, as well as accommodation for tuberculous and special cases, on a valuable site adjoining the hospital and presented to it for this purpose.

**Royal Hospital for Diseases of the Chest, City Road, E.C.** — This hospital is in a serious financial position, for several years expenditure having considerably exceeded income. The annual expenditure averages £7,500; the more or less reliable income amounts to £3,700, leaving an annual deficit of £3,800. The result is that the hospital is indebted to its bankers to the extent of £8,000, and during the last four years there has been a falling off in annual subscriptions of upwards of £400. The outlook is therefore serious, and the committee appeal urgently for funds. A number of soldiers suffering from diseases of the chest are under treatment.

**Royal London Ophthalmic Hospital (Moorfields Eye Hospital), City Road, E.C.**—One physician, one surgeon, the *x*-ray officer, the bacteriologist, the third house surgeon, ten chief clinical assistants, and four clinical assistants are serving with the Forces. In spite of this the work has been carried on, but the strain is severe. The average daily attendances in the out-patient department are over 400, and it will be impossible to continue the efficient working of the hospital if the staff is further reduced. By arrangement with the military authorities twenty beds were set aside for wounded soldiers requiring ophthalmic treatment. Last year 2,137 soldiers were admitted as out-patients and 119 as in-patients. The urgent need of the hospital is an extension with rooms for nurses, for whom the present accommodation is inadequate. For five years in succession the income of the hospital has fallen short of the expenditure.

**Royal National Hospital for Consumption, Ventnor.** Funds are urgently needed for the maintenance of the large number of patients. The annual expenses exceed £15,000, being £1,426 over 1914, by reason of the general increase in prices occasioned by the war. A number of sailors and soldiers suffering from consumption are now under treatment at this hospital. The board would like to erect a special building where an installation of *x*-ray and other apparatus might be placed, also to build a nurses' home. A tower or spire to complete the chapel is much desired. Funds and new annual subscribers for these purposes are earnestly needed.

**Royal National Sanatorium for Consumption, etc., Bournemouth.**—It shows the widespread usefulness of this institution that of the patients admitted in 1915, 417 came from no fewer than twenty-nine different counties in England and two from Wales. Many of the patients were sailors and soldiers; there were also several war refugees from Belgium. But the committee draw attention to the fact that voluntary contributions have diminished, and are £200 less than the previous year, while expenditure has unavoidably increased. [*Continued on p.* 304.

### A YEAR'S WORK IN THE MEDICAL CHARITIES. [*Continued from p.* 299.

### STRATFORD AND EAST-END DISTRICT.

**Comprising Bethnal Green, Tower Hamlets, West Ham, Whitechapel, Hackney, Stepney, Limehouse, Poplar, and the East.**

| No. of Beds. | No. of Beds Daily Occupied. | HOSPITALS. | In-patients | Out-patient Attendances. | Total Expenditure. | Income. Charitable. | Proprietary. | Patients' Payments. | Total Income. | Legacies not included in preceding column. |
|---|---|---|---|---|---|---|---|---|---|---|
| | | | | | £ | £ | £ | £ | £ | £ |
| 184 | 168 | German ... ... ... ... | 1,687 | 32,752 | 14,026 | 3,520 | 5,222 | 1,129 | 9,871 | 404 |
| 1,172 | 874 | London ... ... ... ... | 18,100 | 523,403 | 160,330 | 67,985 | 33,828 | 16,020 | 117,833 | 8,860 |
| 50 | 38 | Mildmay Mission Hospital ... | 584 | 33,959 | 4,298 | 2,929 | 1,222 | 182 | 4,333 | 70 |
| 30 | 21 | Mildmay Memorial ... ... | 251 | ... | 2,211 | 823 | 1,086 | 203 | 2,112 | 10 |
| 115 | 80 | Poplar ... ... ... ... | 1,642 | 71,509 | 10,759 | 12,835 | 3,171 | 958 | 16,964 | 2,894 |
| 46 | 38 | Walthamstow, &c. ... ... | 433 | 11,685 | 2,401 | 2,200 | 330 | 86 | 2,616 | 458 |
| 148 | 124 | West Ham, &c. ... ... ... | 1,747 | 131,449 | 14,373 | 8,776 | 1,554 | 971 | 11,301 | 30 |
| 175 | 156 | City of London for Dis. of the Chest | 782 | 28,710 | 14,505 | 9,047 | 1,454 | 2,177 | 12,678 | 1,145 |
| 126 | 112 | East London for Children ... | 1,538 | 66,093 | 11,468 | 8,996 | 1,832 | ... | 10,828 | 1,201 |
| 43 | 38 | St. Mary's, Plaistow, for Children | 614 | 33,007 | 4,133 | 3,550 | 627 | 113 | 4,290 | 133 |
| 33 | 21 | East End Mothers' Home ... | 549 | 23,587 | 2,826 | 1,526 | 1,378 | 244 | 3,148 | 100 |
| 20 | 13 | Plaistow Maternity ... ... | 342 | 120,729 | 766 | 281 | ... | 153 | 434 | ... |
| 26 | 23 | Canning Town Cottage ... ... | 295 | 13,735 | 2,341 | 1,200 | 462 | 422 | 2,084 | 4 |
| 14 | 10 | Passmore Edwards Cottage, T'lb'ry | 186 | 2,599 | 953 | 668 | 116 | 24 | 808 | ... |
| 25 | 17 | East Ham Cottage ... ... | 167 | 15,468 | 1,164 | 1,312 | 159 | 325 | 1,796 | ... |
| 2,207 | 1,733 | | 28,917 | 1,108,685 | 246,554 | 125,648 | 52,441 | 23,007 | 201,096 | 15,309 |
| | | DISPENSARIES. | | | | | | | | |
| ... | ... | All Saints', Buxton Street ... | ... | 2,467 | 105 | 95 | ... | ... | 95 | ... |
| ... | ... | Eastern ... ... ... ... | ... | 29,779 | 874 | 171 | 309 | 381 | 861 | ... |
| ... | ... | London ... ... ... ... | ... | 2,195 | 419 | 81 | 246 | ... | 327 | 100 |
| ... | ... | Mildmay Medical Mission ... | ... | 5,790 | 220 | 61 | ... | 34 | 95 | ... |
| ... | ... | Queen Adelaide's ... ... ... | ... | 10,555 | 559 | 395 | 356 | ... | 751 | ... |
| ... | ... | Whitechapel Provident ... ... | ... | 5,935 | 341 | 124 | ... | 260 | 384 | ... |
| 2,207 | 1,733 | | 28,917 | 1,165,406 | 249,072 | 126,575 | 53,352 | 23,682 | 203,609 | 15,409 |

### KENSINGTON AND WEST DISTRICT.

**Comprising Kensington, Paddington, Bayswater, Kilburn, Chelsea, Brompton, Fulham, Hammersmith, Chiswick, Brentford, Acton, Ealing, &c.**

| No. of Beds. | No. of Beds Daily Occupied. | HOSPITALS. | In-patients | Out-patient Attendances. | Total Expenditure. | Income. Charitable. | Proprietary. | Patients' Payments. | Total Income. | Legacies not included in preceding column. |
|---|---|---|---|---|---|---|---|---|---|---|
| | | | | | £ | £ | £ | £ | £ | £ |
| 349 | 312 | St. George's ... ... ... | 4,503 | 102,503 | 44,910 | 14,979 | 14,279 | 3,646 | 32,904 | 14,868 |
| 298 | 247 | St. Mary's ... ... ... ... | 3,703 | 103,103 | 34,444 | 12,757 | 10,686 | 1,093 | 24,536 | 63,235 |
| 177 | 170 | West London ... ... ... | 2,562 | 147,687 | 20,215 | 13,112 | 1,857 | 2,508 | 17,477 | 122 |
| 482 | 453 | Hospital for Consumption ... | 1,757 | 27,670 | 45,319 | 22,565 | 4,139 | 14,462 | 41,166 | 5,139 |
| 40 | 36 | Belgrave, for Children ... ... | 745 | 32,115 | 4,472 | 3,073 | 824 | 106 | 4,003 | ... |
| 82 | 79 | Cheyne, for Sick & Incurable Chldn | 56 | ... | 4,447 | 1,497 | 1,460 | 559 | 3,516 | 258 |
| 18 | 13 | Kensington, General ... ... | 233 | 31,907 | 2,379 | 2,311 | 30 | 330 | 2,671 | 65 |
| 13 | 9 | Kensington, for Children ... | 153 | 8,552 | 1,125 | 876 | 220 | ... | 1,096 | 500 |
| 46 | 35 | Paddington Green, for Children | 661 | 45,631 | 5,705 | 3,102 | 2,325 | 302 | 5,729 | 100 |
| 184 | 142 | Victoria, for Children ... ... | 1,276 | 65,629 | 11,973 | 7,700 | 2,649 | 2,373 | 12,722 | 520 |
| 50 | 49 | Chelsea, for Women ... ... | 879 | 9,409 | 7,050 | 5,402 | 919 | 950 | 7,271 | 250 |
| 131 | 93 | Cancer ... ... ... ... | 778 | 16,154 | 19,749 | 2,961 | 8,591 | ... | 11,552 | 24,557 |
| 145 | 110 | Female Lock ... ... ... | 623 | ... | 7,012 | 5,731 | 47 | 1,874 | 7,652 | 2,470 |
| 24 | 22 | Banstead Surgical Home ... | 93 | ... | 958 | 367 | 47 | 465 | 879 | ... |
| 30 | 27 | Acton Cottage ... ... ... | 311 | 7,316 | 1,661 | 1,148 | 195 | 369 | 1,712 | ... |
| 77 | 39 | Ealing Cottage ... ... ... | 671 | ... | 3,149 | 2,194 | 251 | 858 | 3,303 | 600 |
| 16 | 10 | Epsom and Ewell Cottage ... | 220 | ... | 906 | 411 | 69 | 271 | 751 | ... |
| 27 | 22 | Hounslow Cottage ... ... | 282 | 5,002 | 1,352 | 780 | 201 | 609 | 1,590 | ... |
| 40 | 36 | Reigate and Redhill Cottage ... | 655 | ... | 2,794 | 2,149 | 277 | 248 | 2,674 | 100 |
| 24 | 20 | Teddington Cottage ... ... | 302 | ... | 1,279 | 1,112 | 145 | 56 | 1,313 | 264 |
| 26 | 22 | Wimbledon Cottage ... ... | 406 | 136 | 1,640 | 1,297 | 197 | 749 | 2,243 | ... |
| 35 | 30 | Wimbledon, Nelson Hospital ... | 486 | 640 | 2,253 | 1,183 | 243 | 537 | 1,963 | 1,193 |
| 2,314 | 1,976 | | 21,355 | 603,454 | 224,792 | 106,707 | 49,651 | 32,365 | 188,723 | 114,241 |
| | | DISPENSARIES. | | | | | | | | |
| ... | ... | Kilburn, Maida Vale ... ... | ... | 4,625 | 365 | 293 | 29 | ... | 322 | ... |
| ... | ... | Kilburn Provident ... ... | ... | 8,574 | 1,098 | 100 | 15 | 993 | 1,108 | ... |
| ... | ... | Notting Hill Provident ... ... | ... | 4,746 | 496 | 80 | 26 | 396 | 502 | ... |
| ... | ... | Paddington Provident ... ... | ... | 4,675 | 285 | 119 | 2 | 164 | 285 | ... |
| ... | ... | Royal Pimlico Provident... ... | ... | 8,190 | 490 | 155 | 116 | 60 | 331 | ... |
| ... | ... | Westbourne Provident ... ... | ... | 2,097 | 229 | 44 | 5 | 178 | 227 | ... |
| 2,314 | 1,976 | | 21,355 | 636,361 | 227,755 | 107,498 | 49,844 | 34,156 | 191,498 | 114,241 |

### NEWINGTON AND SOUTH DISTRICT.

Comprising Battersea, Wandsworth, Tooting, Balham, Streatham, Brixton, Lambet h, Newington, Southwark, Bermondsey, Camberwell, Greenwich, Deptford, Lewisham, Blackheath, Woolwich, &c.

| No. of Beds. | No. of Beds Daily Occupied. | HOSPITALS. | In-patients. | Out-patient Attendances. | Total Expenditure. | Income. | | | Total Income. | Legacies not included in preceding column. |
|---|---|---|---|---|---|---|---|---|---|---|
| | | | | | | Charitable. | Proprietary. | Patients' Payments. | | |
| | | | | | £ | £ | £ | £ | £ | £ |
| 644 | 557 | Guy's ... ... ... ... | 9,384 | 457,877 | 91,152 | 17,455 | 48,555 | 5,297 | 71,307 | 7,340 |
| 803 | 653 | King's College ... ... ... | 12,898 | 89,395 | 74,942 | 9,445 | 8,320 | 47,425 | 65,190 | 1,528 |
| 18 | 13 | Phillips' Memorial Homœopathic | 143 | 2,241 | 1,426 | 482 | 357 | 488 | 1,327 | 45 |
| 66 | 66 | Miller ... ... ... ... | 916 | 83,616 | 8,750 | 8,892 | 971 | 604 | 10,467 | 75 |
| 60 | 44 | St. John's, Lewisham ... ... | 464 | 9,164 | 4,038 | 1,732 | 883 | 1,281 | 3,896 | 530 |
| 983 | 680 | St. Thomas's ... ... ... | 9,995 | 246,652 | 97,299 | 8,016 | 69,017 | 17,405 | 94,438 | 2,336 |
| 345 | 215 | Seamen's ... ... ... ... | 2,603 | 58,419 | 23,719 | 15,465 | 5,078 | 6,132 | 26,675 | 1,945 |
| 60 | 50 | Bolingbroke Hospital ... ... | 781 | 26,168 | 6,616 | 2,291 | 1,172 | 1,050 | 4,513 | 125 |
| 76 | 54 | Evelina, for Children ... ... | 1,086 | 44,810 | 8,248 | 2,465 | 4,634 | 294 | 7,393 | 979 |
| 57 | 41 | Home for Sick Children... ... | 380 | 6,369 | 2,594 | 1,292 | 278 | 406 | 1,976 | 725 |
| 36 | 30 | General Lying-In... ... ... | 811 | 9,570 | 5,546 | 2,319 | 3,157 | ... | 5,476 | ... |
| 50 | 32 | Clapham Maternity & Dispensary | 660 | 5,088 | 2,565 | 359 | 912 | 1,201 | 2,472 | ... |
| 106 | 76 | Royal Waterloo ... ... ... | 1,112 | 22,068 | 8,327 | 6,515 | 1,530 | ... | 8,045 | 19 |
| 42 | 33 | Royal Eye ... ... ... ... | 567 | 64,446 | 5,554 | 2,250 | 1,086 | 1,355 | 4,691 | 2,542 |
| 28 | 16 | Beckenham Cottage ... ... | 285 | 576 | 1,475 | 1,070 | 51 | 343 | 1,464 | 200 |
| 26 | 20 | Blackheath Cottage ... ... | 191 | 4,046 | 2,056 | 849 | 313 | 641 | 1,803 | 500 |
| 42 | 22 | Bromley Cottage ... ... ... | 374 | 397 | 2,258 | 1,107 | 685 | 411 | 2,203 | ... |
| 28 | 17 | Chislehurst, &c., Cottage ... | 247 | ... | 1,695 | 681 | 198 | 500 | 1,379 | 90 |
| 22 | 12 | Eltham Cottage ... ... ... | 198 | ... | 1,097 | 707 | 124 | 282 | 1,113 | 200 |
| 12 | 7 | Sidcup Cottage ... ... ... | 148 | 166 | 653 | 490 | 46 | 234 | 770 | ... |
| 34 | 24 | Livingstone Cottage ... ... | 319 | ... | 1,495 | 1,396 | 96 | 142 | 1,634 | ... |
| 23 | 16 | Victoria Hospital, Kingston ... | 296 | ... | 1,333 | 754 | 243 | 285 | 1,282 | ... |
| 14 | 10 | British Home for Mothers and Babies ... ... ... | 185 | 2,400 | 1,124 | 708 | 227 | 392 | 1,327 | ... |
| 3,575 | 2,688 | | 44,043 | 1,133,468 | 353,962 | 86,740 | 147,933 | 86,168 | 320,841 | 19,179 |
| | | **DISPENSARIES.** | | | | | | | | |
| ... | ... | Battersea Provident ... ... | ... | 88,000 | 3,634 | 168 | 46 | 3,437 | 3,651 | ... |
| ... | ... | Brixton, &c. ... ... ... | ... | 11,925 | 596 | 409 | 42 | 75 | 526 | ... |
| ... | ... | Camberwell Provident ... ... | ... | 60,217 | 1,102 | 204 | 203 | 631 | 1,038 | ... |
| ... | ... | Clapham ... ... ... ... | ... | 7,309 | 417 | 187 | 83 | 179 | 449 | ... |
| ... | ... | East Dulwich Provident ... | ... | 16,053 | 1,014 | 96 | 24 | 867 | 987 | ... |
| ... | ... | Forest Hill Provident ... ... | ... | 13,215 | 525 | 170 | 16 | 314 | 500 | ... |
| ... | ... | Greenwich Provident ... ... | ... | 21,348 | 443 | 46 | 10 | 397 | 453 | ... |
| ... | ... | Royal South London ... ... | ... | 3,980 | 429 | 333 | 22 | 2 | 357 | ... |
| ... | ... | South Lambeth, &c. ... ... | ... | 2,305 | 345 | 133 | 59 | 50 | 242 | ... |
| ... | ... | Wandsworth Common ... ... | ... | 1,624 | 189 | 12 | ... | 177 | 189 | ... |
| ... | ... | Woolwich, &c., Provident ... | ... | 13,604 | 673 | 39 | ... | 643 | 682 | ... |
| 3,575 | 2,688 | | 44,043 | 1,373,048 | 363,329 | 88,537 | 148,438 | 92,940 | 329,915 | 19,179 |

### THE MEDICAL CHARITIES OF LONDON.—A SUMMARY OF THE WORK DONE IN 1915.

It will be seen from the following summary that *One hundred and sixty-four thousand and seventy-two* patients were admitted into the Voluntary Hospitals and Medical Charities of London during the year 1915, and that the attendances in the Out-Patient Departments and Dispensaries numbered *Five million seven hundred and seventy-one thousand three hundred and thirty-two*, at a cost of *£1,529,656*. The Ordinary Income amounted to *£1,371,529*, leaving a deficiency of *£158,127* on the year's work. The Legacies received in 1915 amounted to *£234,410*.

| No. of Beds. | No of Beds Daily Occupied. | HOSPITALS AND DISPENSARIES. | In-patients. | Out-patient Attendances. | Total Expenditure. | Income. | | | Total Income. | Legacies not included in preceding column. |
|---|---|---|---|---|---|---|---|---|---|---|
| | | | | | | Charitable. | Proprietary. | Patients' Payments. | | |
| | | | | | £ | £ | £ | £ | £ | £ |
| 3,575 | 2,688 | Newington and South District ... | 44,043 | 1,373,048 | 363,329 | 88,537 | 148,438 | 92,940 | 329,915 | 19,179 |
| 1,749 | 1,323 | City and East Central District... | 20,094 | 882,241 | 205,904 | 83,144 | 91,264 | 25,422 | 199,830 | 15,138 |
| 1,067 | 825 | Westminster District ... ... | 10,110 | 437,934 | 120,185 | 79,912 | 19,077 | 30,954 | 129,943 | 8,906 |
| 2,232 | 1,817 | St. Marylebone, &c., District ... | 24,951 | 703,909 | 221,632 | 98,439 | 48,604 | 38,134 | 185,177 | 42,328 |
| 2,314 | 1,976 | Kensington and West District ... | 21,355 | 636,361 | 227,755 | 107,498 | 49,844 | 34,156 | 191,498 | 114,241 |
| 1,592 | 1,284 | Islington & North-West District | 14,602 | 572,433 | 141,779 | 78,171 | 23,974 | 29,412 | 131,557 | 19,209 |
| 2,207 | 1,733 | Stratford and East-End District | 28,917 | 1,165,406 | 249,072 | 126,575 | 53,352 | 23,682 | 203,609 | 15,409 |
| 14,736 | 11,646 | | 164,072 | 5,771,332 | 1,529,656 | 662,276 | 434,553 | 274,700 | 1,371,529 | 234,410 |

### Hospitals and their Special Needs.

[Continued from p. 301.

**Royal Westminster Ophthalmic Hospital, King William Street, Strand, W.C.**—Increased support is needed to meet the growing claims upon this institution. Expenses of maintenance tend constantly to increase with the advance of therapeutical science and appliances, and are still further enhanced by the special exigencies of the moment, and although the committee's appeals for support have been responded to, the ordinary income was not sufficient to meet the expenses of the charity by £341. Ten beds have been reserved for the use of invalided soldiers suffering from injuries or diseases of the eye, and the hospital is daily attending to numbers of recruits for the new Armies and Territorial Forces temporarily below the standard of fitness required. This year marks the centenary of the hospital (1816-1916).

**St. George's Hospital, S.W.**—The ordinary expenditure in 1915 exceeded the ordinary income by £11,296, which had to be met by the sale of stock, by an overdraft at the bank, and by using up legacies received. The cost of provisions and drugs, etc., is much higher than in 1915. While ministering to the sick poor as usual, a number of beds have been allocated for the reception and treatment of the sick and wounded from the Front, of whom 68 sailors and 377 soldiers were admitted during the year. The house committee most urgently appeal to the charitable to help them, so that they can maintain the hospital in its full efficiency.

**St. John's Hospital for Diseases of the Skin, 49 Leicester Square, W.C.**—Secretary, George A. Arnaudin.—For over fifty years St. John's has been doing steady and excellent work, and at the present time some 300 in-patients are treated annually, and in round figures 8,000 out-patients, making a total of about 40,000 attendances. The hospital needs special help for renewing its electrical plant—cost, about £250. Since the beginning of the war nearly 1,000 sailors, soldiers, and refugees have been treated free of charge.

**St. John's Hospital, Morden Hill, Lewisham, S.E.**—During the past year the number of beds, which had been increased from forty-nine to sixty to accommodate wounded and sick soldiers, remained at the latter figure. Five hundred and eleven in-patients were treated, of whom 199 were soldiers. The number of attendances on out-patients—confined to those requiring surgical, bacteriological, or *x*-ray treatment—was 9,164, as against 7,114 in 1914, the increase being attributable to a certain extent to the attendances on soldiers upon their being inoculated before proceeding on service. Notwithstanding the great increase in the price of all provisions and the phenomenal rise in the price of drugs, the average total cost of each in-patient per week was 1s. 11d. less and the average cost of each out-patient attendance nearly 3d. less than in 1914, showing that the utmost economy, consistent with efficiency, was practised. The upkeep of the hospital is practically dependent on donations and subscriptions.

**St. Mark's Hospital, City Road, E.C.**—Before the war the committee had decided, at a cost of over £1,000, to build a special women's ward for cancer patients, also additional accommodation for the nursing staff, but unless the charitable public see their way to support this effort to cope with the ever-increasing cases of rectal cancer this scheme must become at present impossible. Twelve beds have been reserved for wounded soldiers.

**St. Mary's Hospital, Paddington, W.**—This hospital has 305 beds, and its work costs £35,000 a year. It is mainly dependent on voluntary contributions. Distinguished among hospitals for the services it is rendering to the country in the present terrible crisis, St. Mary's is yet suffering heavily financially through the war. In addition to nursing sick and wounded soldiers from the Front, St. Mary's has manufactured and supplied not only our own but all the Allied Armies with anti-typhoid and other vaccines. Several million doses have been supplied, and this work has been acknowledged by the Army Council as "of great practical value to the troops in the field as well as to the State." Similar testimony has been borne by the Belgian, French, Russian, and Serbian authorities. It is common knowledge that the Allied Armies in the Western battlefield have enjoyed a degree of immunity from typhoid fever which has deprived war of one of its terrors, and this is due in a great degree to work done in St. Mary's Hospital.

**St. Monica's Home Hospital for Sick Children, 16 Brondesbury Park, N.W.**—For over forty years this hospital has carried on its beneficent work, and until the past two years free from debt. Recently, however, there has been a considerable falling-off in the receipts, from donations chiefly, and at the end of 1915 there was a deficiency of £391.

**St. Peter's Hospital for Stone, etc., Henrietta Street, W.C.**—During the past year, to comply with the requirements of the L.C.C., two external iron staircases, as a means of escape in case of fire, have been erected at a cost of £640. King Edward's Hospital Fund contributed the sum of £200, but the rest had to be met by a loan. Decreasing contributions and increasing costs of commodities make the task of financing the hospital one of great anxiety. Twelve beds were reserved for the use of wounded soldiers, though these have not so far been occupied; but patients have been treated to enable them to join the Army or proceed to the Front, and many of the staff and nurses have joined the Forces.

**Samaritan Free Hospital for Women, Marylebone Road, N.W.**—This hospital has now entered its seventieth year. Many of the patients are the wives, mothers, or dependants of our sailors and soldiers on active service. Many of them are sufferers from cancer, others are young women whom early treatment will fit for healthy motherhood. When the question of infant mortality has become a national problem, the restoration to health of the young women is of vital importance. The committee of management specially plead for funds to assist them in reducing the loan to the bankers of £3,500 and to endow a ward for special cases.

**Seamen's Hospital Society ("Dreadnought" and Albert Dock Hospitals), Greenwich, S.E.**—Although by the action of the enemy every merchant seaman is become a combatant, he is still dependent upon public generosity for care and treatment when

[Continued on p. 306.

### THE VOLUNTARY HOSPITALS' BUDGET.

In these anxious times our hospitals and kindred institutions must not be allowed to suffer any degree of want that may cripple their efficiency. The health of every man, woman, and child is as precious to the nation as to the individual, and an organisation such as the voluntary system, which does so much to secure a high level of health to the community, both by administering directly to the patients and by training the doctors and nurses who look after those who do not use the hospitals, must be maintained. To support these institutions now is an act of charity, and at the same time an act of patriotism of a most useful kind. The hospitals appeal especially to us at the present time when we know what they are doing for our brave wounded sailors and soldiers, to whom particularly those of us who are unable to fight feel such a debt of gratitude; and while they are, in most cases by means of extra bed accommodation, performing this duty, the utmost is also done for the sick and suffering poor. And all this work is carried out under great difficulty; staffs of doctors, nurses, and all ranks are depleted, those who are left to work do so, in most cases, during much longer hours and at greater pressure than in normal times, while the expensive nature of special supplies, such as drugs, dressings, and instruments, in conjunction with the increased cost of food and daily necessaries, is a heavy drain upon the institutions.

The ordinary expenditure of the Metropolitan Voluntary Hospitals and Dispensaries ordinarily increases by about £40,000 a year. In 1912 it was about £1,255,000, in 1913 about £1,295,000, and in 1914 about £1,334,000. No one can foretell what it will be in 1916, but it is sure to be greatly in excess of the last total quoted. To meet this heavy expenditure the hospitals and dispensaries have to rely upon three main sources of revenue.

First and most important of these are the gifts of the charitable, for which we are now appealing, such as subscriptions, donations, entertainments, grants raised by King Edward's Hospital Fund and the Hospital Sunday and Saturday Funds, and other receipts of a voluntary character. This source of revenue, together with the payments made by patients towards cost of maintenance or medicines (the latter about 7 per cent. of the total revenue), supplies about half the amount required for the upkeep of the institutions, and may be taken as worth about £800,000 a year. But to reach that figure a renewal of the generosity of friends in the past is necessary, for new friends but take the place of those who are lost by death or changed circumstances. Expenses are not likely to be less than £1,500,000, and the £800,000 above mentioned would bring in under 11s. of every £1 required. Secondly, there is the income from investments and property. The value of this is increasing by about £10,000 to £15,000 a year, and should continue to do so. The amount received in 1913 was £350,000, and in 1914 £366,000, so that in 1915 we may look for £375,000, which would amount to 5s. towards each sovereign required.

These two fairly stable sources of income may be expected to provide 16s. in the pound, but the other 4s., or one-fifth of the total requirements, must be looked for as coming from a more precarious source; indeed, the only remaining one from which revenue can be derived, viz., the gifts left to hospitals in the shape of legacies. These vary in amount from year to year; in a period of ten years they have fallen as low as £186,000, and reach as high as £451,000, the last figure being for 1914. Curiously, two indifferent years often follow an unusually good year, and if this condition applies to 1916 we may very likely not obtain the requisite £300,000, or 4s. in the sovereign. But in any case legacies should be regarded as extraordinary income, and should be utilised to assist in meeting the expenditure required for rebuilding, for improvements requisite to keep pace with the rapid march of science, or for extension where overcrowding has become apparent, all of which items of extraordinary expenditure have now to be met by special appeals to those who already gave largely.

But extraordinary expenditure finds no place in the present budget. Ordinarily about a quarter of a million a year is spent in this way, but nearly all schemes of rebuilding, enlargement, or alterations are postponed until peace-time.

In conclusion, having shown that such support is necessary, may we ask the old supporters of the Metropolitan Hospitals and Dispensaries to give this year, as nearly as possible, as liberally as they have done in the past? and we call upon those who have not hitherto given to remember what the voluntary hospitals have done, and are doing, for the outside community, to recognise under what difficulties they are working at present, and to become welcome friends and supporters at a time when it is possible that these charities may have to suffer in consequence of the heavy calls being made in so many ways upon the purse of the public.

### INCOME.

Table Showing Income of the Metropolitan Hospitals and Dispensaries in the Ten Years 1905 to 1914.

| Year | From the Living | | From the Dead | | | | Total Income |
|---|---|---|---|---|---|---|---|
| | Subscriptions, Donations, Patients' payments, etc. | Percentage of Total | Investments | Percentage of Total | Legacies | Percentage of Total | |
| | £ | | | | £ | | £ |
| 1905 | 610,130 | 49 | 264.790 | 21 | 373 914 | 30 | 1.248 834 |
| 1906 | 635.377 | 58 | 272.704 | 25 | 186.284 | 17 | 1.094 365 |
| 1907 | 653,320 | 48 | 296,218 | 22 | 407.918 | 30 | 1.357,456 |
| 1908 | 712 461* | 56 | 290.491 | 23 | 265.493 | 21 | 1.268.445 |
| 1909 | 701.458 | 57 | 294,206 | 24 | 240 523 | 19 | 1,236.187 |
| 1910 | 703,841 | 50 | 309 320 | 22 | 394.478 | 28 | 1.407.639 |
| 1911 | 738 622 | 53 | 329 253 | 24 | 322 949 | 23 | 1.390.824 |
| 1912 | 741,439 | 55 | 338 425 | 25 | 272.090 | 20 | 1,351 954 |
| 1913 | 845.908* | 52 | 349 295 | 22 | 424 339 | 26 | 1.619.542 |
| 1914 | 801.239 | 49 | 366,841 | 23 | 4 51,569 | 28 | 1,619,649 |

* Includes £72,565 and £103,970, the amounts received by the London Hospital in 1908 and 1913 respectively as the result of its Quinquennial Appeals in those years.

### Hospitals and their Special Needs.

[*Continued from p. 304.*

wounded, sick, or injured. Subscriptions, donations, and legacies are urgently needed to maintain the work of the hospitals. Over 1,000 naval ratings have been received and about 100 additional beds are in constant use.

**Teddington and Hampton Wick Cottage Hospital** is in need of additional subscribers to meet deficits in connection with the ordinary work of the hospital. There is a constant burden of about £200, which hampers the work considerably and seems impossible to remove.

**Victoria Hospital for Children, Chelsea.**—Since the outbreak of war many annual subscriptions have been withdrawn and reduced, and the income has in consequence been reduced by over £300. The two wards given up during 1915 for sick and wounded soldiers are again occupied by children. The cost of maintenance has greatly increased, and without additional financial help there will be a large deficit at the end of the year. The hospital contains 104 beds, with sixty-four beds for convalescent patients at the seaside. One thousand two hundred and thirty-three patients were treated in hospital in 1915, and 491 received convalescent treatment. The total cost of maintenance was £11,674, all of which has to be raised from voluntary sources, with the exception of £2,273 derived from dividends.

**West End Hospital, Welbeck Street, W.**—Owing to the war a much-needed rebuilding scheme has been temporarily suspended. New subscribers are wanted, as the work which before the war cost about £6,200 per annum has already risen to over £8,000, and is continually increasing. The income from invested property amounts to £1,300, and there is a debt of £3,000 to wipe out. In addition to the usual work for civilians (which has not been curtailed), temporary wards for nerve-injured and nerve-wrecked soldiers have been opened. Many of these are very bad and tedious cases, and require lengthy treatment. The War Office contributes towards their maintenance, but this contribution has to be largely supplemented from the hospital funds, and the outlay in preparing this new accommodation has also to be met.

**West London Hospital, Hammersmith.**—The hospital serves an immense district, having an area of forty-five square miles and a population of over half-a-million. No fewer than 40,000 sick and poor in- and out-patients are treated every year. The annual expenditure amounts to £19,500, and the assured income from endowments is £600 only. Practically £19,000 has to be raised every year from voluntary sources. One effect of the war is the difficulty experienced in raising money. This, and the greatly enhanced cost of all commodities, render the task of the board of management in finding the necessary funds a very anxious one. The hospital is doing its best to help in the present crisis, and on the outbreak of war accommodation for 100 sick and wounded soldiers from overseas was placed at the disposal of the War Office. From October 1914 to April 1916 754 soldiers have been admitted as in-patients and 1,645 as out-patients, with most successful results. Subscriptions and donations towards carrying on the West London Hospital's vast and increasing work will be most gratefully received and acknowledged.

**Westminster Hospital, London, S.W.**—The expenditure necessary to the maintenance of this old-established hospital in London, supported by voluntary contributions, shows a deficit of £3,417 over income for the first quarter of the current year. If, therefore, this charity is to maintain efficiently its good work, additional support of the most generous character is immediately required. The removal of the hospital to a more commodious site is in contemplation, and as this will involve a very large expenditure, liberal contributions to the "Removal Fund" are very earnestly solicited. The total number of beds is 213, and upon the outbreak of war seventy-five of these beds were placed at the disposal of the War Office for the treatment of sick and wounded soldiers from the Front, and this number has since been increased to eighty-six. Up to the present date 614 British and 52 Belgian soldiers have been treated.

**Wimbledon Hospital.**—An appeal is made for more annual subscribers. The subscriptions last year were only £637; the upkeep of the hospital requires £1,700. There are many indications that the demands upon the hospital are increasing, and must continue to increase, for in the neighbourhood of Raynes Park, in proximity to the hospital, a very large poor population is springing up, and it will require all the interest that can be secured to enable the executive committee to meet the demands. There are twenty beds for wounded soldiers who are sent direct from the Front. Contributions should be sent to the Hon. Secretary, Mr. A. T. Burton, at the Hospital, Thurstan Road, Wimbledon.

### OTHER INSTITUTIONS AND THEIR SPECIAL NEEDS.

**Convalescent Home for Poor Children, St. Leonards-on-Sea.**—Contributions are needed to the general fund to enable the work amongst the poor children, most of whose fathers are sailors or soldiers, to be carried on.

**Metropolitan Convalescent Institution, 14 Victoria Street, S.W.**—The maintenance of the four homes at Walton, Broadstairs, Bexhill-on-Sea, and Little Common, Bexhill, containing 586 beds in all, costs £14,000 a year, for nearly the whole of which the institution is dependent upon voluntary contributions. The board of management appeal very earnestly for further annual subscriptions and donations.

**St. Mary's Convalescent Home, Birchington-on-Sea.** Pecuniary help is needed in consequence of increased cost of food and fuel. Widows of soldiers, with their children, and other women have been enabled to benefit by the bracing air of Birchington. Donations will be gladly received by Lady Fremantle, the Treasurer, at Sloane Terrace Mansions, S.W.

**St. Michael's Convalescent Home, Westgate-on-Sea.** The special and immediate need is certainly more support. The home has suffered much from loss of subscriptions, and finds it more and more difficult to meet the cost of maintenance. Belgian and English soldiers have been received.

## Figures and Tables

**Figure f1:**
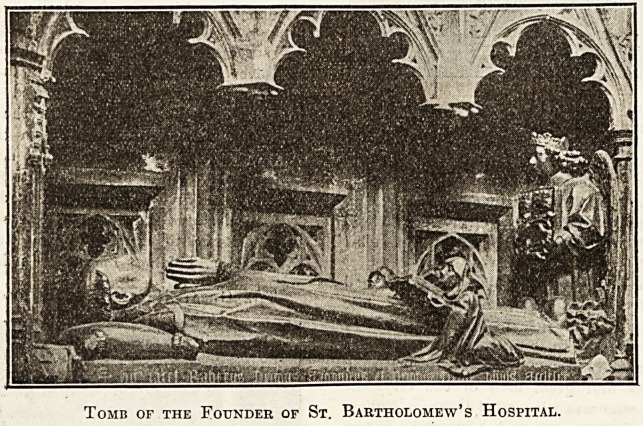


**Figure f2:**
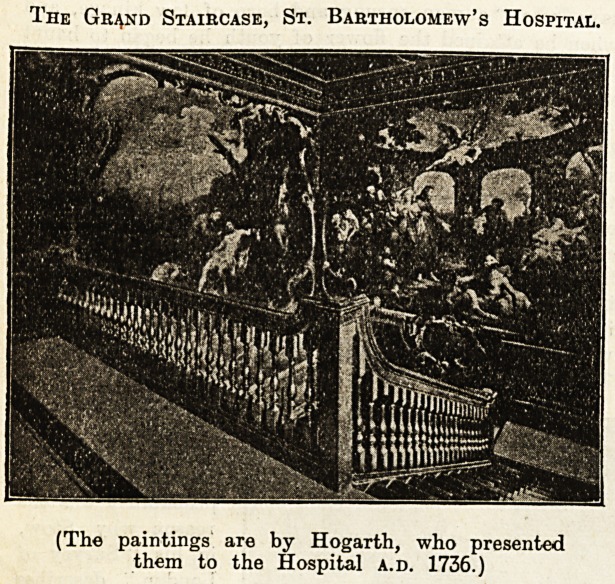


**Figure f3:**
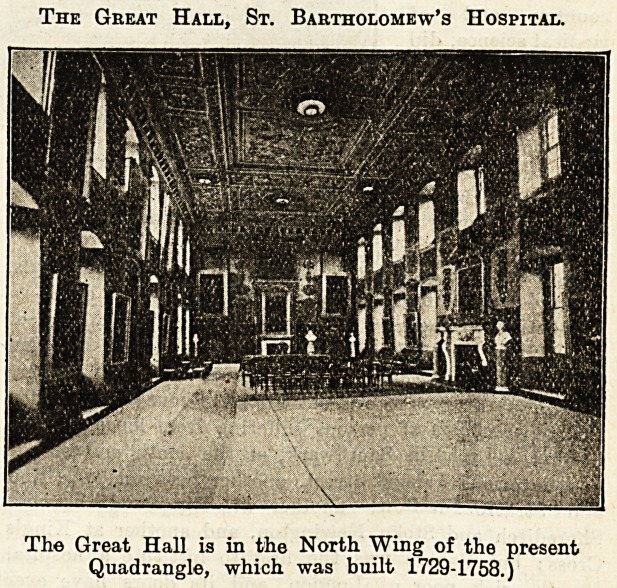


**Figure f4:**
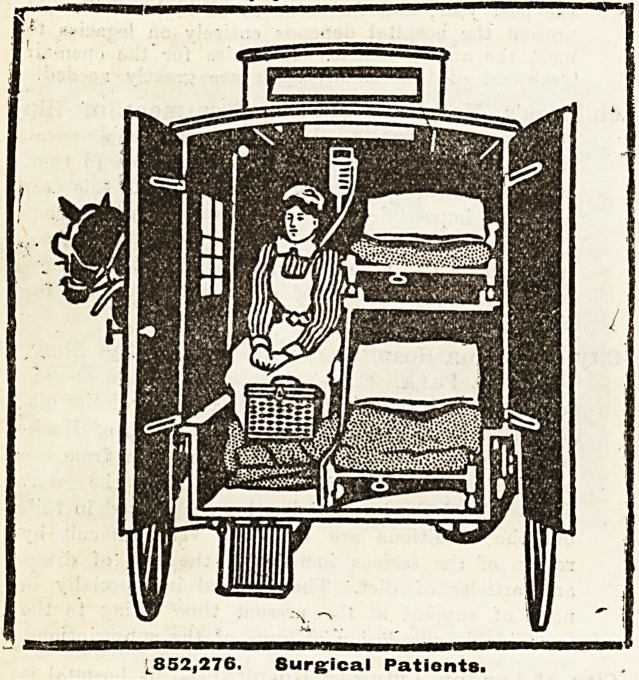


**Figure f5:**
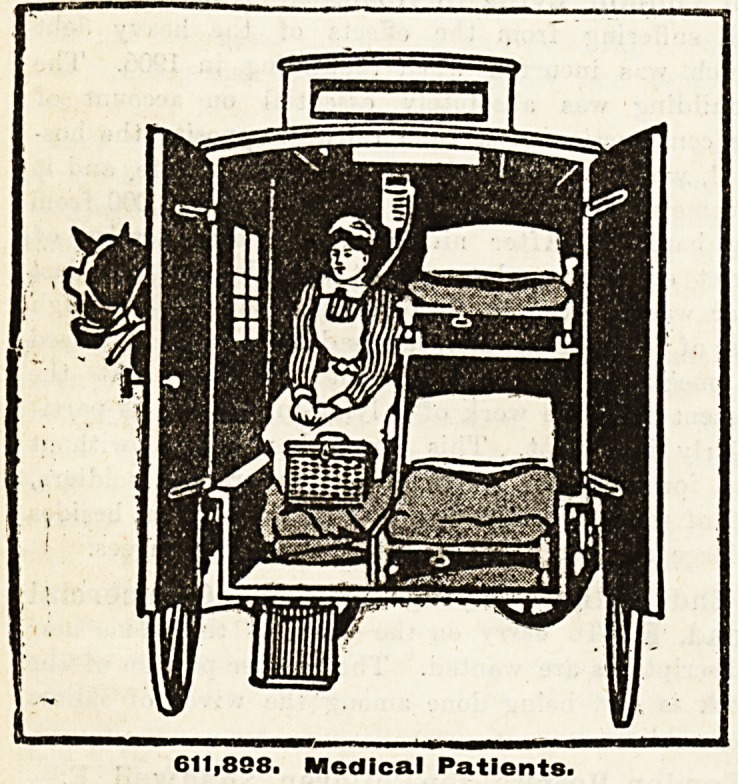


**Figure f6:**
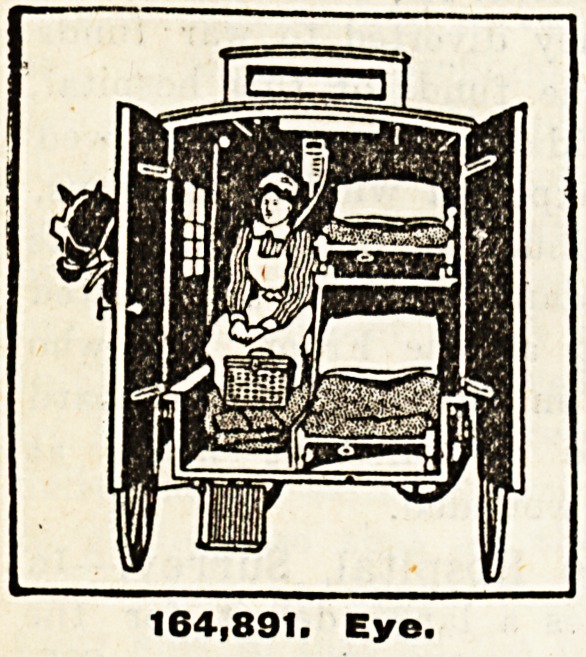


**Figure f7:**
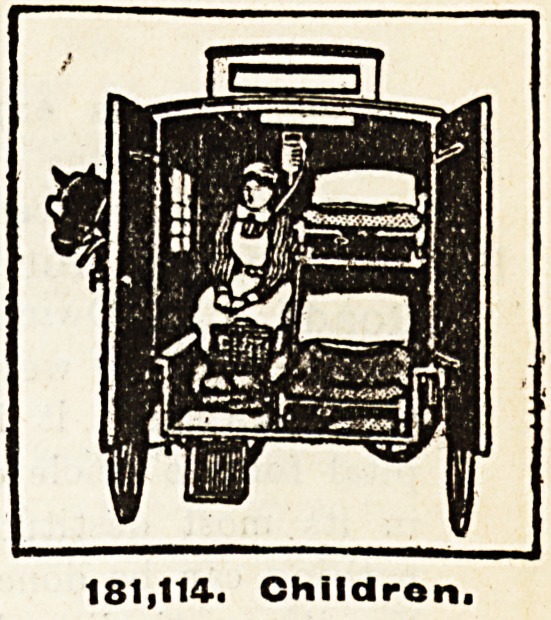


**Figure f8:**
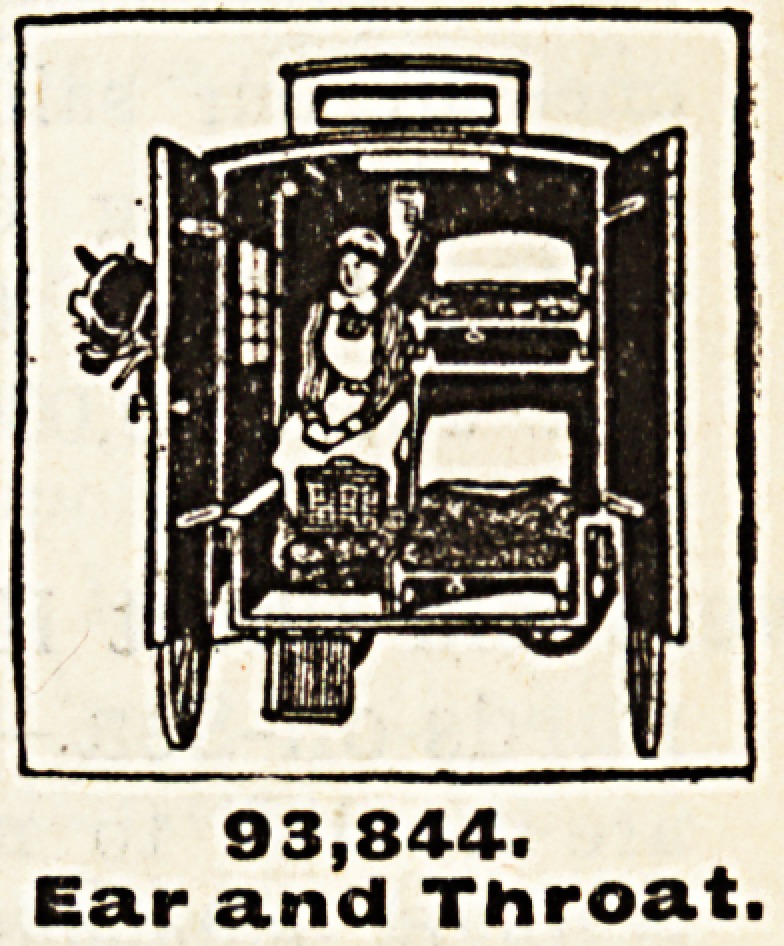


**Figure f9:**
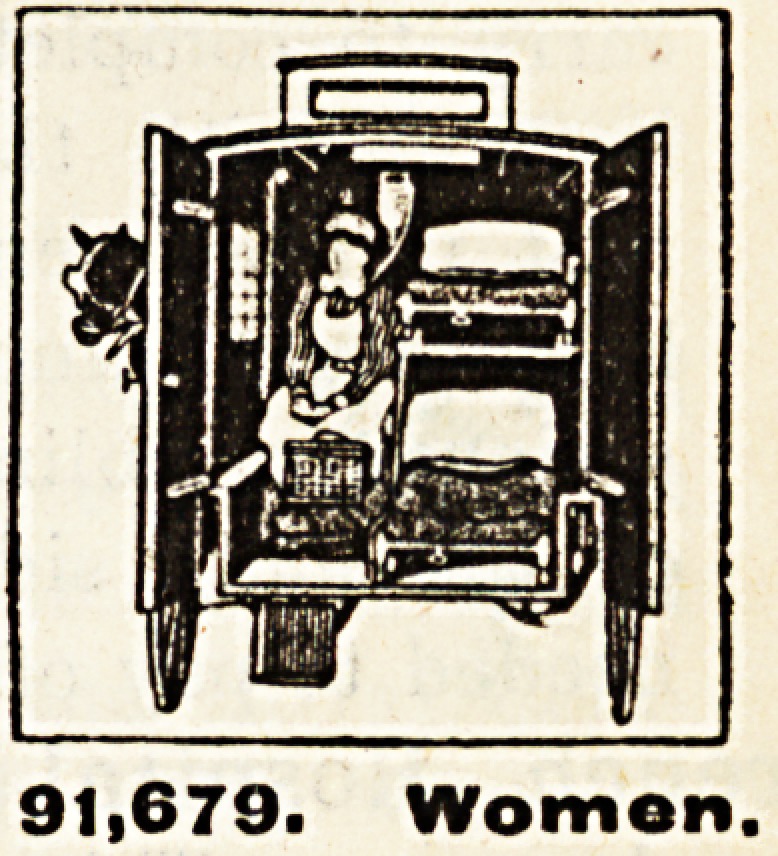


**Figure f10:**
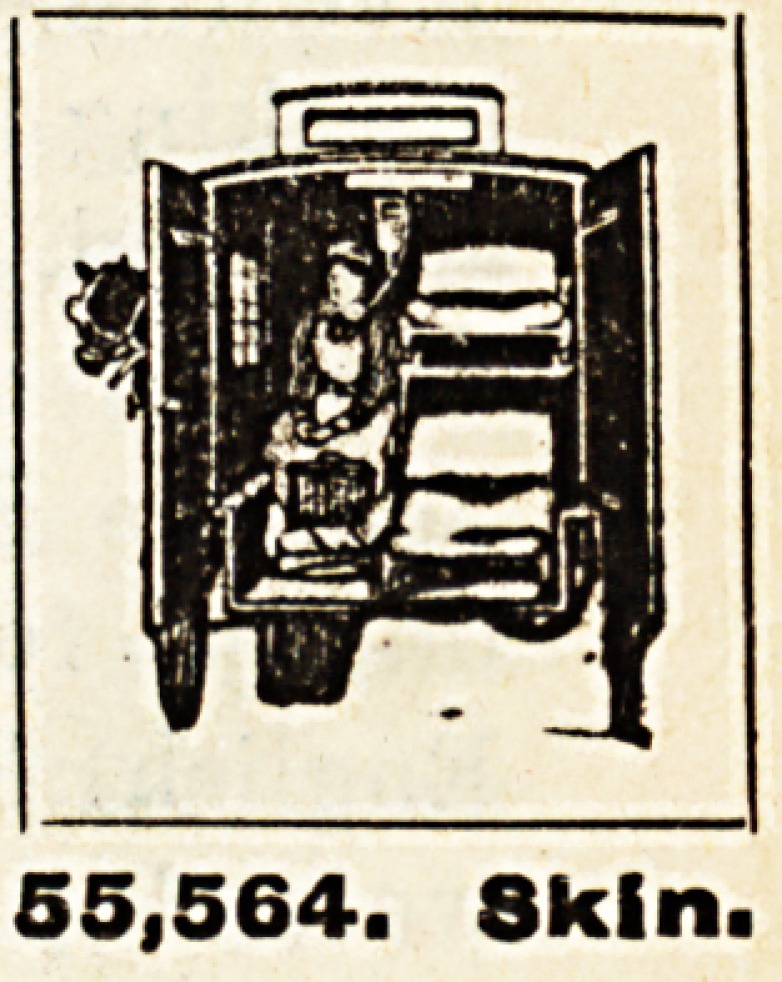


**Figure f11:**
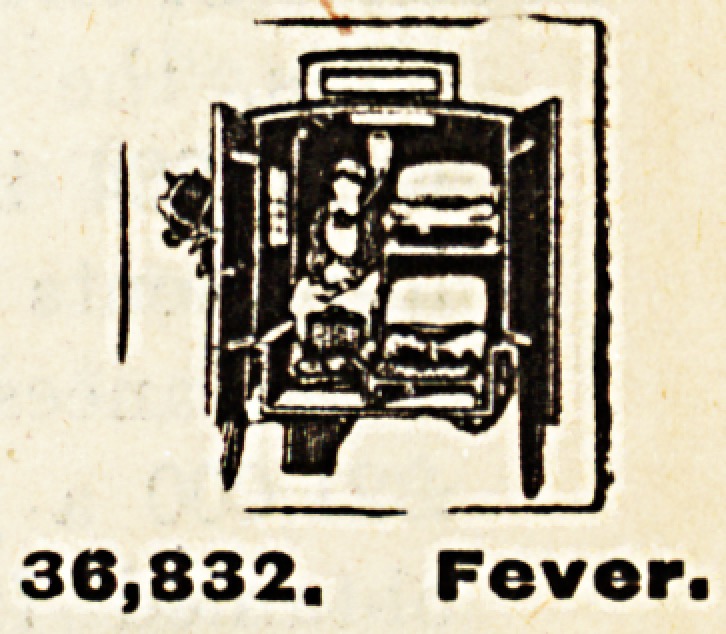


**Figure f12:**
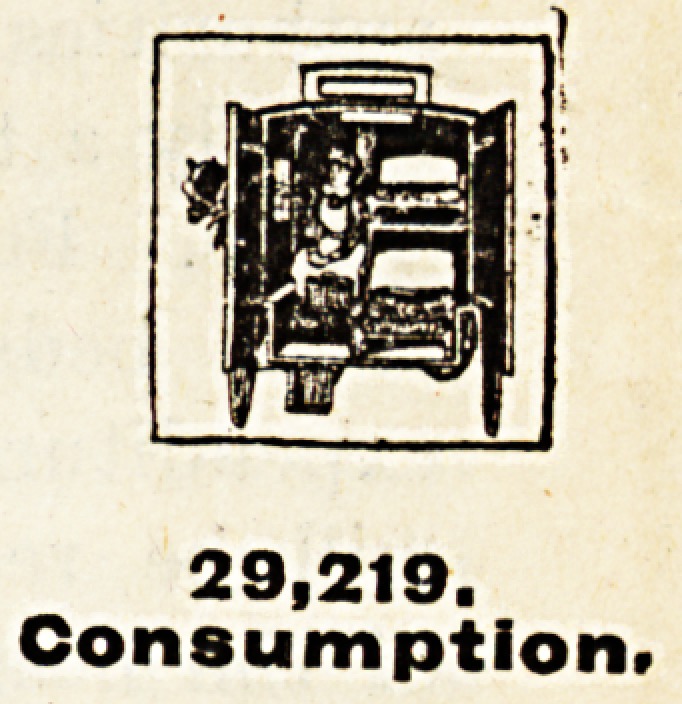


**Figure f13:**